# Chaotic billiards optimized hybrid transformer and XGBoost model for robust and sustainable time series forecasting

**DOI:** 10.1038/s41598-025-10641-7

**Published:** 2025-07-17

**Authors:** Reham H. Mohammed, Asmaa Mohamed El-saieed

**Affiliations:** 1 Department of Electrical Computer and Control Engineering, Faculty of Engineering, Suez Canal University, Ismailia, 41522 Egypt; 2 Department of Communication and Electronics, Mansoura High Institute of Engineering and technology, Mansoura, Egypt

**Keywords:** Energy science and technology, Engineering

## Abstract

Accurate wind speed forecasting plays a key role in supporting renewable energy systems, improving flight safety, and enhancing weather prediction. However, the variability and non-stationary nature of wind patterns make reliable forecasting a difficult task. To address these issues, this research proposes a hybrid approach that combines Wavelet Transform (WT) decomposition, an Encoder-Decoder Transformer, and XGBoost in an ensemble setup. To fine-tune model parameters efficiently, the method incorporates the Chaotic Billiards Optimizer (CBO) alongside the Adam optimizer. In this framework, WT helps break down wind speed signals into different frequency bands, capturing both short-term changes and long-term behavior. The Transformer model focuses on learning complex time-based dependencies, while XGBoost adds robustness to the final predictions by reducing overfitting and improving generalization. The model forecasts wind speed values on an hourly basis, up to 24 h ahead. The use of CBO ensures efficient convergence with minimal parameter tuning, making the model suitable for large-scale datasets compared to conventional optimizers, including Adam, Particle Swarm Optimization (PSO) and Genetic Algorithms (GA). When tested on real wind speed data collected from Egypt via the Open-Meteo platform, the proposed model showed strong performance. It achieved a Mean Absolute Error (MAE) of 0.0218, Mean Squared Error (MSE) of 0.0008, and Root Mean Squared Error (RMSE) of 0.0290, along with an R² score of 0.9625, MAPE of 11.97%, and an Explained Variance Score (EVS) of 0.9521. These results were better than those from models like Linear Regression, SVR, LSTM, and even standalone Transformer and XGBoost. Overall, this hybrid method provides a reliable and efficient solution for wind speed forecasting in the context of sustainable energy planning.

**Supplementary Information:**

The online version contains supplementary material available at 10.1038/s41598-025-10641-7.

## Introduction

This section is structured to present the research background, review relevant literature, and outline the study’s objectives and innovations.

### Research background

The rapid evolution of modern industries has significantly increased the global demand for energy. Simultaneously, environmental concerns and the finite availability of fossil fuels—such as coal, oil, and natural gas—have underscored the urgency for clean, renewable energy alternatives. Among these, wind energy is considered one of the most promising options due to its plentiful availability, environmental friendliness, and cost efficiency^1^.

Despite these advantages, several challenges hinder the full exploitation of wind energy. The most notable of these challenges is the intermittency and variability of wind, which directly impacts the reliability of wind energy forecasting and, consequently, the quality of power supplied to the grid^2^. The inherent instability of wind speed makes it difficult to accurately determine the optimal amount of electricity to deliver to the distribution network. This volatility can destabilize power systems and hamper efficient energy dispatching. Therefore, precise and dependable wind speed forecasting is essential for maintaining grid stability and supporting large-scale renewable integration.

The results of this study provide detailed insights that contribute to the improvement of wind turbine control systems, enhance load tracking performance, and optimize wind power distribution within energy grids.

### Literature review

Recent studies in wind speed forecasting have employed a wide array of models, which can be broadly classified into single models and hybrid models^3^.

####  Researches using single models

Single-model approaches include physical models, spatio-temporal correlation (STC) models, statistical methods, and machine learning (ML) techniques^4^.

Physical models are grounded in meteorological laws and can yield reliable long-term forecasts. However, they are limited in short-term applications due to data latency issues^5^.

Spatio-temporal correlation (STC) models depend on synchronized data from multiple weather stations, which can produce reliable forecasts, but they often suffer from high data acquisition costs and time-consuming preprocessing^6^.

Statistical models, such as Auto-Regressive Integrated Moving Average (ARIMA), have proven effective in short-term forecasting using historical wind data^7^. However, they typically assume linearity, which is often not the case in real wind patterns.

Machine learning models—including support vector machines, decision trees, and artificial neural networks—have been proposed to tackle wind data’s nonlinearity and volatility^8^. Yet, ML models often risk converging to local optima, which limits their generalization capabilities.

####  Researches using hybrid models

Given the instability and complexity of wind speed data, hybrid models have been widely adopted to enhance both forecasting accuracy and model robustness. Most hybrid models follow a “decomposition–optimization–forecasting” framework^9^.

Decomposition involves breaking down the raw wind signal into simpler, more stable sub-series. Techniques such as Empirical Mode Decomposition (EMD)^10^, Variational Mode Decomposition (VMD)^11^, and hybrid methods like CEEMDAN-EWT^12^ and SSA-VMD^13^ have shown strong performance in this area.

Optimization fine-tunes the forecasting model’s parameters. Techniques like Backtracking Search Algorithm (BSA)^14^, Chicken Swarm Optimization (CSO)^13^, and Genetic Algorithms (GA)^15^ have been used for either optimizing model structures or selecting relevant features. Multi-objective optimization (MOO) strategies have also emerged to simultaneously balance accuracy, complexity, and training cost^9^.

Forecasting models, such as ARIMA^16^, VAR^10^, and NARX neural networks^17^, are employed to process each decomposed sub-series. With the rise of big data and sensor networks, Deep Learning (DL) has become a prominent approach due to its superior feature extraction capabilities^18^. Notably, LSTM-based models have achieved high accuracy in short-term wind speed prediction tasks^19,20^.

To better highlight the strengths, limitations, and methodological distinctions of various wind speed forecasting approaches, Table 1 presents a comparative summary of the reviewed literature. This overview categorizes each study based on the model type, employed techniques, key advantages, and limitations, providing a clearer understanding of the research landscape and justifying the motivation for the proposed hybrid model.

**Table 1:** Summary of key literature on wind speed forecasting Models.

Model type	Techniques Used	Advantages	Limitations	References
Physical models	Numerical Weather Prediction (NWP)	Accurate for long-term predictions; based on atmospheric physics	Poor short-term accuracy; high computational cost	^3^
Statistical models	ARIMA, VAR	Good for linear data; fast computation	Ineffective for non-linear and non-stationary data	^4,16^
Machine learning	SVM, ANN, Random Forest	Handles non-linearity; adaptable to various data types	Risk of overfitting; requires large datasets	^5,8^
Decomposition methods	EMD, VMD, WT, CEEMDAN-EWT, SSA-VMD	Reduces signal complexity; enhances feature extraction	Requires careful selection of decomposition level and method	^6,9–13^
Optimization algorithms	Genetic Algorithm, Chicken Swarm Optimization, Hybrid BSA, CBO	Enhances model parameter tuning; improves convergence and accuracy	May increase computational complexity	^9,13–15^
Hybrid models	Decomposition + Optimization + ML/Statistical/DL models (e.g., ELM, LSTM, ARIMA)	Combines strengths of multiple models; robust and accurate	Complex architecture; parameter tuning required	^10,12,16,19^
Deep learning	LSTM, Transformer, NARX	Excellent temporal modeling; automatic feature extraction from large datasets	Training-intensive; needs large and high-quality datasets	^18–20^
Ensemble models	XGBoost, Ensemble of ML/DL models	High generalization ability; can handle overfitting and model variance	Requires multiple base learners; may increase training complexity	^15,19^

While existing hybrid models such as EMD-LSTM and VMD-GA-ARIMA have demonstrated improved accuracy through decomposition and optimization techniques, they often suffer from limitations such as suboptimal hyperparameter tuning, inadequate handling of multi-scale temporal features, and a lack of generalization under noisy meteorological conditions. These models typically rely on single decomposition methods or conventional optimizers, which restrict their forecasting robustness and computational efficiency. To address these challenges, our proposed model uniquely integrates Wavelet Transform (WT) for capturing multi-frequency features, an Encoder-Decoder Transformer to learn long-term temporal dependencies, and XGBoost to enhance generalization and reduce overfitting. Furthermore, the use of the Chaotic Billiards Optimizer (CBO) with Adam introduces a novel optimization strategy that improves convergence speed and accuracy across large-scale, real-world wind datasets.

### Objectives and innovations

This study introduces the Chaotic Billiards-Optimized Transformer–XGBoost (CBOTran-XGBoost) hybrid model, integrating signal decomposition (Wavelet Transform), deep learning (Transformer with positional encoding), and ensemble learning (XGBoost) into a unified framework. It also incorporates Chaotic Billiards Optimization (CBO) with Adam for efficient parameter tuning.

Key contributions of this research are summarized as follows:

1. Integration of Wavelet Transform (WT) with Positional Encoding in the Transformer architecture to better capture multi-scale temporal patterns in time series data.
2. Hybrid optimization using Adam and CBO, ensuring both local convergence and global exploration.
3. Ensemble learning via XGBoost, which enhances generalization and reduces the risk of overfitting.
4. An autoregressive Transformer decoder that sequentially generates wind speed predictions for improved forecast consistency.
5. Implementation of key-value caching during inference to reduce computational overhead and increase efficiency.

## The Proposed CBOTran-XGBoost Hybrid Model Architecture

The proposed architecture is designed to address the challenges of wind speed forecasting by integrating three key modules: the Data Preprocessing and Decomposition Module, the Optimization Module, and the Forecasting Module. Each module plays a critical role in enhancing the model’s ability to capture complex temporal patterns, optimize training, and improve generalization.

**Fig. 1:**
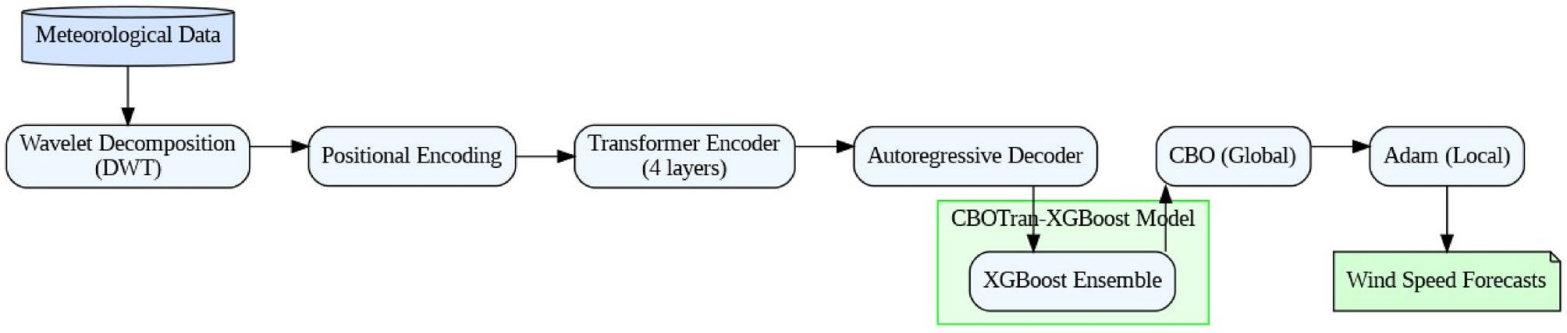
Conceptual map of the proposed hybrid wind speed forecasting model.

Figure 1 illustrate a conceptual map of a proposed hybrid model for wind speed forecasting. Meteorological data is first decomposed using Discrete Wavelet Transform to extract key features, followed by Positional Encoding to retain temporal information. A four-layer Transformer Encoder captures long-term dependencies, and an Autoregressive Decoder generates initial predictions. These are refined using the CBOTRAN-XGBoost model, which integrates Chaotic Butterfly Optimization (CBO) for global tuning and XGBoost) for regression. Finally, Adam is applied for local optimization, producing accurate Wind Speed Forecasts.

**Fig. 2:**
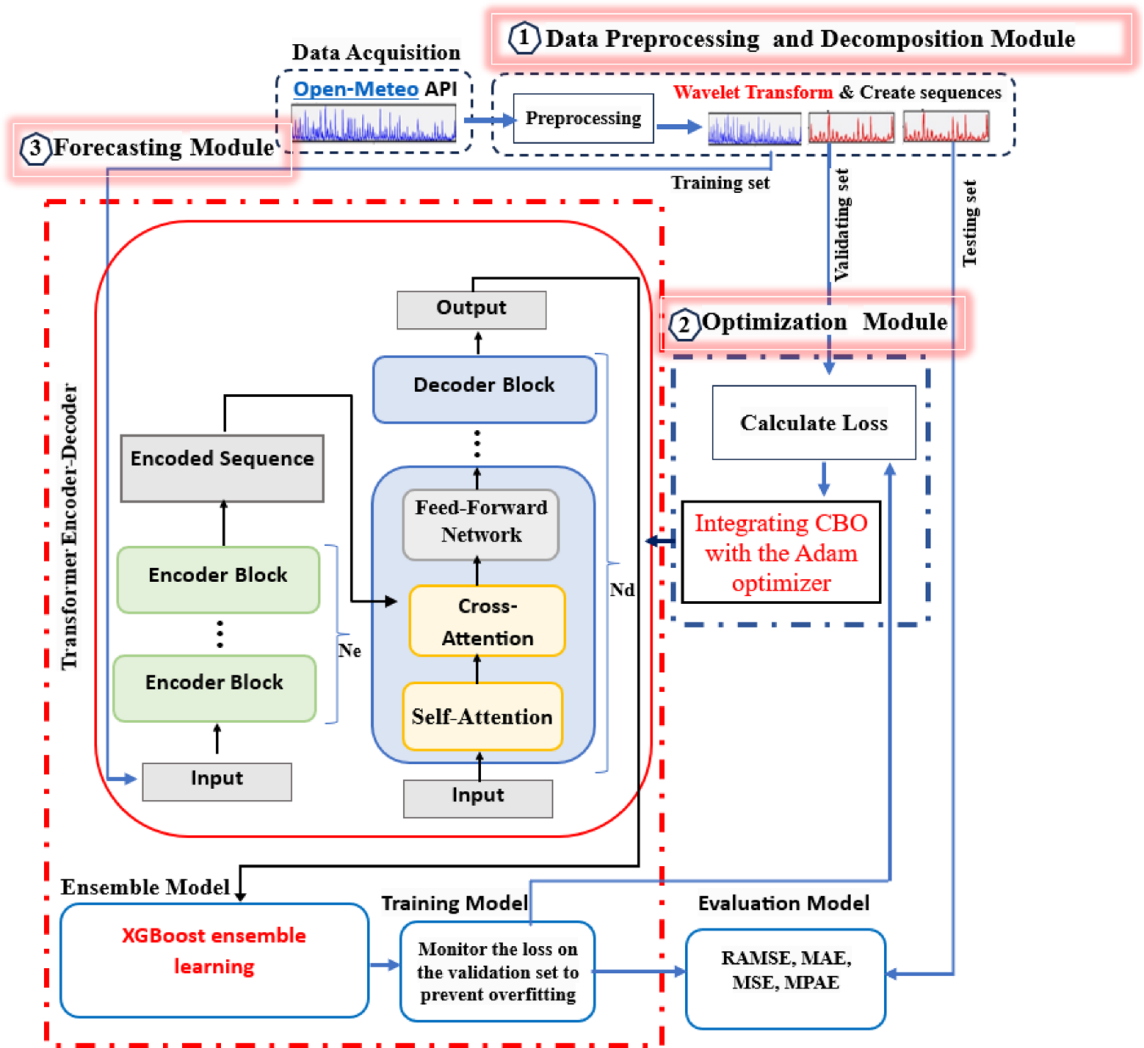
The flowchart of the proposed CBOTran-XGBoost architecture.

The proposed architecture is built on the Transformer framework, which is adapted for time series forecasting tasks. The architecture consists of three interconnected modules as shown in Fig. 2:

I. Data Preprocessing and Decomposition Module: Preprocesses the input wind speed time series data using Discrete Wavelet Transform (DWT) and Positional Encoding to capture multi-scale temporal patterns.
II. Optimization Module: Combines Adam optimization CBO to enhance the training process and improve model convergence.
III. Forecasting Module: Utilizes an Encoder-Decoder Transformer Model for sequence-to-sequence prediction, combined with ensemble models (XGBoost) to improve generalization and robustness.

### Data preprocessing and decomposition module

The Data Preprocessing and Decomposition Module are responsible for preprocessing the input wind speed time series data to extract multi-scale temporal features. Data preprocessing is essential for ensuring the quality and accuracy of the forecasting model. It involves handling missing values, normalizing the dataset, and splitting it into training and testing sets to prepare it for machine learning and deep learning models^9^. The dataset used in this study includes hourly meteorological records, such as temperature, humidity, pressure, wind speed, wind direction, soil temperature, and solar radiation, which are critical for understanding wind speed patterns. To prepare the data, timestamps are converted into a uniform datetime format for proper indexing and temporal analysis. Feature normalization, such as Min-Max scaling, is applied to ensure numerical stability and prevent large-scale features from dominating the model. An initial assessment revealed that less than 1.5% of the dataset contained missing values. To maintain the integrity of temporal sequences and avoid introducing bias through synthetic data, we adopted a complete-case analysis strategy. Specifically, rows with missing entries were removed using the dropna() method in Python. This method was chosen over imputation techniques such as linear interpolation or k-nearest neighbors (KNN), given the low level of missingness and the importance of preserving sequential data continuity for accurate forecasting^21^. The dataset is split into 80% training and 20% testing sets to evaluate the model’s generalization capability. To model wind speed forecasting as a supervised learning task, a lag window of 24 h was used, capturing past 24 hourly observations of input features. This autoregressive setup allows the model to learn temporal dependencies. All data were normalized using Min-Max Scaling, and missing values were dropped. A walk-forward validation strategy is employed, where the model is trained on past data and tested on future time steps. This approach mimics real-world forecasting scenarios, enhancing the reliability of predictions. The Decomposition module integrates two key components: DWT and Positional Encoding.

#### DWT

Wind speed data is often non-stationary, meaning it contains trends, seasonality, and irregular fluctuations. The DWT is selected due to its effectiveness in signal processing and feature extraction^9^. DWT is particularly well-suited for analyzing non-stationary time series data.

The process begins by decomposing the input wind speed sequence into multiple frequency components using DWT, which allows the model to detect both short-term variations (high-frequency elements) and long-term patterns (low-frequency elements). DWT employs a mother wavelet function, such as Daubechies or Haar, to break down the signal into approximation coefficients, representing the overall trend, and detail coefficients, capturing finer details and fluctuations. Daubechies (db4) and Haar wavelets were chosen for DWT due to their effectiveness in capturing both smooth trends and sharp changes in wind speed data. Compared to Symlet, they showed lower RMSE in preliminary tests and better preserved transient features. The decomposition level *N* = 3 was selected empirically to balance noise reduction and information retention, with higher levels offering minimal performance gain and lower levels failing to isolate key patterns.The resulting set of approximation and detail coefficients from the DWT decomposition are then fed into the positional encoding of Transformer model. One of the key advantages of this approach is that DWT allows the model to capture multi-scale temporal patterns, which are crucial for accurate wind speed forecasting. By separating the input signal into different frequency components, the model can effectively focus on both short-term and long-term dependencies. Additionally, DWT is computationally efficient and provides a compact representation of the signal, rendering it ideal for real-time applications^12^.

Let’s assume our dataset contains the following variables: Wt represents the wind speed at a given time t, Tt denotes the temperature at time t, Ht signifies the humidity at time t, and Pt indicates the pressure at time t. The dataset also includes past values:

1 $$\:{X}_{t}=[{W}_{t-3},{W}_{t-2},{W}_{t-1},{T}_{t,}{H}_{t},\:{P}_{t}]$$

The target variable:

2 $$\:{Y}_{t}={W}_{t}$$

We decompose Wt​ using WT into different frequency components^22^:

3 $$\:{W}_{t}=\sum\:_{j=1}^{N}{(D}_{j}\left(t\right)+{A}_{j}\left(t\right))$$

Where Wt​ represents the original signal at time t, The summation$\:\sum\:_{\text{j}=1}^{\text{N}}{\text{D}}_{\text{j}}\left(\text{t}\right)$ accounts for the detail components across multiple levels j (from 1 to N), Aj​(t) is the approximation component at a certain level j, This equation indicates that the initial signal is restored by adding up the approximation component and the sum of detail components.

Now, the input dataset becomes:

4 $$\:{X}_{t}=[{D}_{t-3},{D}_{t-2},{D}_{t-1},{{A}_{t-1},T}_{t,}{H}_{t},\:{P}_{t}]$$

Using Min-Max Normalization^23^:

5 $$\:{X}^{{\prime\:}}=\frac{X-{X}_{min}}{{X}_{max}-{X}_{min}}$$

where:

- Xmax​, Xmin​ are max/min values in each column.

####  Positional encoding

The architecture of the Transformer model does not inherently capture the sequential order of time steps in the input data. Positional encoding is introduced to the input data to enable the model to recognize the sequential order of time steps. The process involves applying sinusoidal positional encoding, a widely used technique in Transformer models, to the input data before it is processed by the Transformer. The positional encoding is defined^23^ as:

6 $$\:{PE}_{pos,2i}=\text{s}\text{i}\text{n}\left(\frac{pos}{{10000}^{2i/d}}\right)$$

7 $$\:{PE}_{pos,2i+1}=\text{c}\text{o}\text{s}\left(\frac{pos}{{10000}^{2i/d}}\right)$$

This encoding method incorporates positional information by using sinusoidal functions, where pos represents the position of the time step, i denotes the dimension, and d corresponds to the dimensionality of the input data. This approach offers several advantages, where the positional encoding enables the model to differentiate between time steps and effectively capture temporal dependencies within the sequence. Additionally, sinusoidal encoding is particularly advantageous as it allows the model to generalize well to sequences of varying lengths, enhancing its flexibility and robustness in handling diverse data inputs.

Final input embedding^24^ is defined as:

8 $$\:{\text{X}}_{\text{i}\text{n}\text{p}\text{u}\text{t}}={\text{X}}^{{\prime\:}}+\text{P}\text{E}$$

###  Forecasting module

The Forecasting Module serves as the central component of the proposed framework, responsible for generating accurate wind speed predictions. It is composed of two primary components: the Encoder-Decoder Transformer Model and Ensemble Models (XGBoost).

#### Encoder-Decoder transformer model

The Encoder-Decoder Transformer Model is used for sequence-to-sequence prediction, where the encoder processes the historical wind speed sequence, and the decoder predicts future wind speed values in an autoregressive manner^23– 24^. Based on the results of the hyperparameter tuning process (see Table 2), the final Transformer configuration includes 4 encoder layers and 4 decoder layers, each utilizing 4 attention heads. This setup was selected to balance model complexity, training efficiency, and forecasting performance. The encoder processes the input sequence using multi-head self-attention mechanisms and feed-forward layers to extract temporal dependencies, while the decoder sequentially generates predictions by attending to both the encoder output and previously generated outputs.

##### Encoder block (Historical data Processing)

The encoder processes the historical wind speed sequence, which has been decomposed and enhanced with positional encoding, to extract high-level temporal features. This is accomplished by employing multi-head self-attention mechanisms in conjunction with feed-forward neural networks, which work together to capture complex patterns and dependencies in the data. The encoder is constructed from multiple Transformer blocks, with each block containing a Multi-Head Attention (MHA) module and a Feed-Forward Network (FFN) module. These components enable the encoder to effectively analyze and transform the input sequence, facilitating the extraction of meaningful features for subsequent stages of the model.

**Fig. 3:**
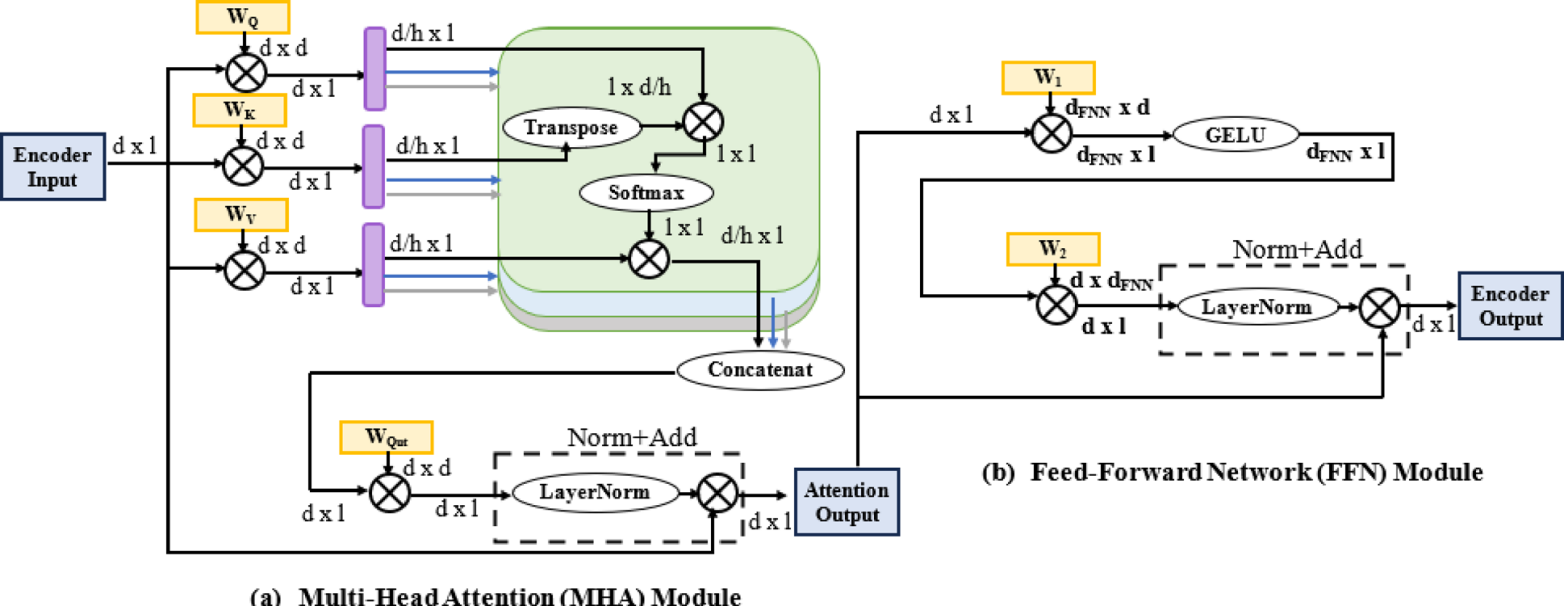
Encoder block architecture (**a**) MHA (**b**) FNN.

A Transformer architecture consists of multiple encoder blocks /decoder blocks, with each block containing a MHA module and a FFN module as shown in Fig. 3. Both modules are followed by layer normalization and a residual connection (Add & Norm). The input to each block is a matrix of size* d* ×* l*, where:

- *l* represents the number of time steps in the input sequence (e.g., historical wind speed data).
- *d* is the dimensionality of each time step’s feature representation.

In the context of wind speed forecasting, a “token” corresponds to a time step in the historical wind speed sequence, and the model processes these tokens to extract temporal patterns.

A) Multi-head attention (MHA):

The MHA module serves as the central element of the Transformer architecture, allowing the model to capture temporal dependencies within the input sequence, as illustrated in Fig. 3a. Here’s how it works in the context of wind speed forecasting:

I. Input projection:

The input sequence (historical wind speed data) is projected through three weight matrices: W^Q^ (query), W^K^ (key), and W^V^ (value). These projections generate three activations: query, key, and value. These activations are split into ℎ chunks (attention heads), each with a hidden dimension of d/ℎ. This enables the model to simultaneously attend to multiple temporal patterns. Let $\:{X}_{input}\in\:{R}^{l\times\:d}$ represent the historical wind speed sequence, where l is the sequence length and d is the feature dimension. Each input sequence is projected into three matrices:

9 $$\:\text{Q}={\text{X}}_{\text{i}\text{n}\text{p}\text{u}\text{t}}{\text{W}}^{\text{Q}}\:$$

10 $$\:\text{K}={\text{X}}_{\text{i}\text{n}\text{p}\text{u}\text{t}}{\text{W}}^{\text{K}}\:$$

11 $$\:\text{V}={\text{X}}_{\text{i}\text{n}\text{p}\text{u}\text{t}}{\text{W}}^{\text{v}}\:$$

Where Q is the query matrix, K is the key matrix, V is the value matrix, and WQ, WK, WV $\:\in\:{\text{R}}^{\text{d}\times\:{\text{d}}_{\text{k}}}$ are the learned projection matrix for queries, keys, and values.

II. Attention mechanism:

Within every attention head, the query and key segments are multiplied across the hidden dimension, resulting in an activation matrix with dimensions ×. This matrix represents the relationships between different time steps in the input sequence. The activation matrix undergoes the Softmax operation, which computes attention scores. These scores determine how much each time step should focus on other time steps. The attention scores are then multiplied by the value chunks, producing an activation of size d/ℎ for each head.

12 $$\:\text{A}\text{t}\text{t}\text{e}\text{n}\text{t}\text{i}\text{o}\text{n}\left(\text{Q},\text{K},\text{V}\right)=\text{s}\text{o}\text{f}\text{t}\text{m}\text{a}\text{x}\left(\frac{\text{Q}{\text{K}}^{\text{T}}}{\sqrt{{\text{d}}_{\text{k}}}}\right)\text{V}\:$$

Where dk = d/h​ is the dimension per attention head of the key vectors, and the Softmax function is employed on the scaled dot-product of the queries and keys.

13 $$\:\text{M}\text{u}\text{l}\text{t}\text{i}\text{H}\text{e}\text{a}\text{d}\left(Q,\:K,\:V\right)=Concat\left({head}_{1},{head}_{2}\:,\dots\:\dots\:.,{head}_{h}\right){W}^{o}\:$$

Where h is the number of heads in the multi-head attention, Concat is the concatenation operation, and WO$\:\in\:{\text{R}}^{\text{d}\times\:\text{d}}$ is the Final linear transformation matrix.

Where each head is computed in Equations ($\:14$):

14 $$\:{\text{h}\text{e}\text{a}\text{d}}_{i}=\text{A}\text{t}\text{t}\text{e}\text{n}\text{t}\text{i}\text{o}\text{n}\left(Q{W}_{i}^{Q},K{W}_{i}^{k},\:V{W}_{i}^{V}\right)\:$$

III. Concatenation and output:

The activations from all attention heads are concatenated along the hidden dimension to form a single activation of size d. This concatenated activation is reflected back to the primary dimension via a final linear layer with the weight matrix W out. The output of this layer passes through LayerNorm and is combined with a residual connection, yielding the final output of the MHA module.

15 $$\:Z=\text{L}\text{a}\text{y}\text{e}\text{r}\text{N}\text{o}\text{r}\text{m}\left({X}_{input}+\text{M}\text{u}\text{l}\text{t}\text{i}\text{H}\text{e}\text{a}\text{d}\left(Q,\:K,\:V\right)\right)\:\:$$

B) Feed-Forward Network (FFN) Module.

The FFN module as illustrated in Fig. 3b. consists of two linear layers with a non-linear activation function in between. In the context of wind speed forecasting, the FFN module helps the model learn complex temporal patterns:

I. First Linear Layer:

The input sequence is projected from the hidden dimension d to a higher FFN dimension FFN using the weight matrix W1. Typically, d_FFN_ is chosen to be four times larger than d, resulting in a 4:1 aspect ratio between d_FFN_ and d.

II. Non-linear activation:

A non-linear activation function, such as GELU, is applied to the projected sequence. As a result, the model gains non-linearity, which helps it to represent intricate temporal interactions.

III. Second linear layer:

The sequence is mapped back to the original dimension d through the second linear layer with weight matrix W2.

16 $$\:H=\text{G}\text{E}\text{L}\text{U}\left(Z{W}_{1}+\text{b}1\right)\:$$

17 $$\:FNN=H{W}_{2}\:\:+b2\:$$

18 $$output=\text{L}\text{a}\text{y}\text{e}\text{r}\text{N}\text{o}\text{r}\text{m}\left(Z+FNN\right)$$

C)Non-linear operations in time series prediction.

Non-linear operations such as Softmax, LayerNorm, and GELU play a crucial role in the Transformer architecture. While these operations represent a smaller portion of the overall computational workload compared to linear operations, they are essential for capturing complex temporal patterns in the data.

I. Softmax: Used in the attention mechanism to compute attention scores. It involves computing exponentials, summing them, and normalizing the results, which can be computationally intensive for long sequences.
II. LayerNorm: Normalizes the activations across the hidden dimension, requiring multiple passes over the input data to compute the mean and standard deviation.
III. GELU: A non-linear activation function used in the FFN module to introduce non-linearity into the model.

##### Decoder block (Future prediction generation)

**Fig. 4:**
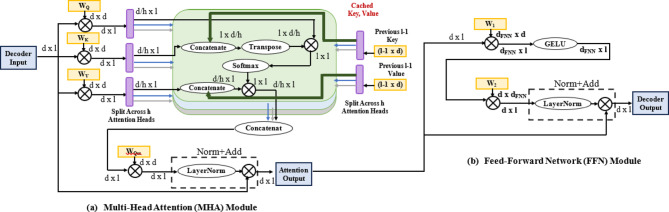
Decoder block architecture (**a**) MHA (**b**) FNN.

The decoder generates future wind speed values step by step, conditioned on both the encoded historical features and the previously predicted values as shown in Fig. 4. Unlike the encoder, which processes the entire historical sequence at once, the decoder operates auto regressively, meaning that each predicted value becomes part of the input for the next prediction step.

A) Autoregressive prediction:

At each time step, the decoder predicts the next wind speed value based on the encoded historical features and the previously predicted values. This allows the model to condition future predictions on its own outputs, ensuring consistency in the forecasted sequence.

We set the autoregressive window size k = 24k = 24 h, corresponding to one full daily cycle. This choice was empirically validated through cross-validation, which showed improved prediction accuracy and stability compared to shorter (e.g., 12-hour) and longer (e.g., 48-hour) windows. The 24-hour window effectively captures diurnal patterns in wind behavior while avoiding over fitting.

The input at time step t is given by:

19 $$\:{Y}_{t}=[{\widehat{y}}_{t-1},{\widehat{y}}_{t-2},\dots\:\dots\:,{\widehat{y}}_{t-k}]$$

Where Yt ​ represents input sequence to the decoder at time t,$\:\:{\widehat{\text{y}}}_{\text{t}-1},{\widehat{\text{y}}}_{\text{t}-2},\dots\:\dots\:,{\widehat{\text{y}}}_{\text{t}-\text{k}}$ are the previously predicted wind speed values, k denotes the number of previous time steps considered.

The decoder function is defined as:

20 $$\:{\widehat{y}}_{t}={f}_{Decoder}\left({Y}_{t},Z\right)\:\:\:\:\:\:\:\:\:\:\:\:\:\:\:\:\:\:\:\:\:\:\:\:\:\:\:\:\:\:\:\:\:\:\:\:\:\:\:\:\:\:\:\:\:\:\:\:\:\:\:\:\:\:\:\:\:\:\:\:\:\:\:\:\:\:\:\:\:\:\:\:\:\:\:\:\:\:\:\:\:\:\:\:\:\:\:\:\:\:\:\:\:\:\:\:\:\:\:\:\:$$

Where Z represents encoded historical data from the encoder, $\:{\widehat{\text{y}}}_{\text{t}}$denotes the predicted wind speed at time t.

B) Attention mechanism:

The decoder uses a combination of self-attention and cross-attention mechanisms as shown in Fig. 4a. The self-attention mechanism allows the decoder to attend to previously predicted values, while the cross-attention mechanism enables it to focus on the encoded historical features from the encoder. This dual attention mechanism ensures that the decoder leverages both historical trends and its own predictions to generate accurate forecasts.

- Self-attention.

Each decoder layer computes self-attention over previously predicted values:

21 $$\:{Q}_{t}={Y}_{t}{W}^{Q}\:\:\:\:\:\:\:\:\:\:\:\:\:\:\:\:\:\:\:\:\:\:\:\:\:\:\:\:\:\:\:\:\:\:\:\:\:\:\:\:\:\:\:\:\:\:\:\:\:\:\:\:\:\:\:\:\:\:\:\:\:\:\:\:\:\:\:\:\:\:\:\:\:\:\:\:\:\:\:\:\:\:\:\:\:\:\:\:\:\:\:\:\:\:\:\:\:\:\:\:\:\:\:\:\:\:\:\:\:\:\:\:\:$$

22 $$\:{K}_{t}={Y}_{t}{W}^{K}\:\:\:\:\:\:\:\:\:\:\:\:\:\:\:\:\:\:\:\:\:\:\:\:\:\:\:\:\:\:\:\:\:\:\:\:\:\:\:\:\:\:\:\:\:\:\:\:\:\:\:\:\:\:\:\:\:\:\:\:\:\:\:\:\:\:\:\:\:\:\:\:\:\:\:\:\:\:\:\:\:\:\:\:\:\:\:\:\:\:\:\:\:\:\:\:\:\:\:\:\:\:\:\:\:\:\:\:\:\:\:\:$$

23 $$\:{V}_{t}={Y}_{t}{W}^{v}\:\:\:\:\:\:\:\:\:\:\:\:\:\:\:\:\:\:\:\:\:\:\:\:\:\:\:\:\:\:\:\:\:\:\:\:\:\:\:\:\:\:\:\:\:\:\:\:\:\:\:\:\:\:\:\:\:\:\:\:\:\:\:\:\:\:\:\:\:\:\:\:\:\:\:\:\:\:\:\:\:\:\:\:\:\:\:\:\:\:\:\:\:\:\:\:\:\:\:\:\:\:\:\:\:\:\:\:\:\:\:\:\:$$

Where Q is the query matrix, K is the key matrix, V is the value matrix, and WQ, WK, WV $\:\in\:{\text{R}}^{\text{d}\times\:{\text{d}}_{\text{k}}}$ are the learned projection matrix for queries, keys, and values.

The masked self-attention score is computed as:

24 $$\:\text{S}\text{e}\text{l}\text{f}-\text{A}\text{t}\text{t}\text{e}\text{n}\left({\text{Y}}_{\text{t}}\right)=\text{s}\text{o}\text{f}\text{t}\text{m}\text{a}\text{x}\left(\frac{{\text{Q}}_{\text{t}}{{\text{K}}_{\text{t}}}^{\text{T}}}{\sqrt{{\text{d}}_{\text{k}}}}+\text{M}\right){\text{V}}_{\text{t}}\:\:\:\:\:\:\:\:\:\:\:\:\:\:\:\:\:\:\:\:\:\:\:\:\:\:\:\:\:\:\:\:\:\:\:\:\:\:\:\:\:\:\:\:\:\:\:\:\:\:\:\:$$

Where dk is dimensionality of the key vectors. M is masking matrix ensuring no access to future time steps.

- Cross-attention with encoded historical data.

The decoder also attends to the encoded sequence Z from the encoder:

25 $$\:{Q}_{t}={Y}_{t}{W}^{Q}\:\:\:\:\:\:\:\:\:\:\:\:\:\:\:\:\:\:\:\:\:\:\:\:\:\:\:\:\:\:\:\:\:\:\:\:\:\:\:\:\:\:\:\:\:\:\:\:\:\:\:\:\:\:\:\:\:\:\:\:\:\:\:\:\:\:\:\:\:\:\:\:\:\:\:\:\:\:\:\:\:\:\:\:\:\:\:\:\:\:\:\:\:\:\:\:\:\:\:\:\:\:\:\:\:\:\:\:\:\:\:\:\:\:\:\:\:\:$$

26 $$\:{K}_{Z}=Z{W}^{K}\:\:\:\:\:\:\:\:\:\:\:\:\:\:\:\:\:\:\:\:\:\:\:\:\:\:\:\:\:\:\:\:\:\:\:\:\:\:\:\:\:\:\:\:\:\:\:\:\:\:\:\:\:\:\:\:\:\:\:\:\:\:\:\:\:\:\:\:\:\:\:\:\:\:\:\:\:\:\:\:\:\:\:\:\:\:\:\:\:\:\:\:\:\:\:\:\:\:\:\:\:\:\:\:\:\:\:\:\:\:\:\:\:\:\:\:\:\:$$

27 $$\:{V}_{Z}=Z{W}^{v}\:\:\:\:\:\:\:\:\:\:\:\:\:\:\:\:\:\:\:\:\:\:\:\:\:\:\:\:\:\:\:\:\:\:\:\:\:\:\:\:\:\:\:\:\:\:\:\:\:\:\:\:\:\:\:\:\:\:\:\:\:\:\:\:\:\:\:\:\:\:\:\:\:\:\:\:\:\:\:\:\:\:\:\:\:\:\:\:\:\:\:\:\:\:\:\:\:\:\:\:\:\:\:\:\:\:\:\:\:\:\:\:\:\:\:\:\:\:\:$$

The cross-attention output is:

28 $$\:\text{C}\text{r}\text{o}\text{s}\text{s}-\text{A}\text{t}\text{t}\text{e}\text{n}\left({\text{Y}}_{\text{t}},\text{Z}\right)=\text{s}\text{o}\text{f}\text{t}\text{m}\text{a}\text{x}\left(\frac{{\text{Q}}_{\text{t}}{{\text{K}}_{\text{Z}}}^{\text{T}}}{\sqrt{{\text{d}}_{\text{k}}}}\right){\text{V}}_{\text{Z}}\:\:\:\:\:\:\:\:\:\:\:\:\:\:\:\:\:\:\:\:\:\:\:\:\:\:\:\:\:\:\:\:\:\:\:\:\:\:\:\:\:\:\:\:\:\:\:\:\:\:\:\:\:\:\:\:\:\:\:$$

This allows the decoder to align its predictions with historical trends captured by the encoder.

c) Feed-forward Network (FNN) and Layer Normalization.

After self-attention and cross-attention, the decoder applies a FNN as appeared in Fig. 4b and layer normalization:

29 $$\:FNN\left({Y}_{t}\right)=\sigma\:({W}_{1}{Y}_{t}+{b}_{1}){W}_{2}\:\:+b2\:\:\:\:\:\:\:\:\:\:\:\:\:\:\:\:\:\:\:\:\:\:\:\:\:\:\:\:\:\:\:\:\:\:\:\:\:\:\:\:\:\:\:\:\:\:\:\:\:\:\:\:\:\:\:\:\:\:\:\:\:\:\:\:\:\:\:\:\:\:\:\:\:\:$$

Where W1​,W2​ and b1 ​,b2​ are learnable parameters, while σ(⋅) represents the activation function, such as ReLU or GELU.

The final decoder output is obtained using LayerNorm:

30 $$\:\widehat{{y}_{t}}=\text{L}\text{a}\text{y}\text{e}\text{r}\text{N}\text{o}\text{r}\text{m}\left({Y}_{t}+FNN\left({Y}_{t}\right)\right)\:\:\:\:\:\:\:\:\:\:\:\:\:\:\:\:\:\:\:\:\:\:\:\:\:\:\:\:\:\:\:\:\:\:\:\:\:\:\:\:\:\:\:\:\:\:\:\:\:\:\:\:\:\:\:\:\:\:\:\:\:\:\:\:\:\:\:\:\:\:\:\:\:\:\:\:\:\:$$

This ensures stable gradients and smooth optimization.

d) Key-value caching:

To optimize the decoding process, the key and value activations from previously predicted time steps are cached and reused. This eliminates the need to recompute these activations at each step, significantly reducing computational overhead^25^.

Instead of computing attention scores at each time step, previous key-value pairs are cached:

31 $$\:{K}^{cache}=\left[{K}_{t-1},{K}_{t}\right]\:{V}^{cache}=\left[{V}_{t-1},{V}_{t}\right]$$

This allows the decoder to reuse past computations, improving efficiency.

Final Prediction Formulation.

The full decoder equation integrating self-attention, cross-attention, feed forward processing, and layer normalization is:

32 $$\:\widehat{{y}_{t}}=\text{L}\text{a}\text{y}\text{e}\text{r}\text{N}\text{o}\text{r}\text{m}\left(\varvec{S}\varvec{e}\varvec{l}\varvec{f}-\varvec{A}\varvec{t}\varvec{t}\varvec{e}\varvec{n}\left({Y}_{t}\right)+\varvec{C}\varvec{r}\varvec{o}\varvec{s}\varvec{s}-\varvec{A}\varvec{t}\varvec{t}\varvec{e}\varvec{n}\left({Y}_{t},Z\right)+FNN\left({Y}_{t}\right)\right)\:$$

This formulation ensures that the decoder effectively leverages historical context through the encoder output (Z), past predictions via self-attention, and efficient computation using key-value caching. As a result, the autoregressive Transformer decoder generates wind speed forecasts step by step while dynamically adapting to both past trends and recent predictions.

#### Ensemble models (XGBoost)

To further enhance the generalization and robustness of the model, we integrate the outputs of the encoder-decoder Transformer model with XGBoost^26^, a highly effective gradient boosting algorithm. This ensemble approach combines the strengths of deep learning and traditional machine learning, resulting in more accurate and reliable wind speed forecasts.

The process begins with the Transformer model generating initial predictions for future wind speed values. These predictions, along with additional relevant features such as meteorological data (e.g., temperature, pressure, humidity), are then fed into the XGBoost model. XGBoost is trained to refine these predictions by leveraging the additional features, producing a final forecast that combines the strengths of both models. This hybrid approach allows the model to capture complex relationships in the data that may not be fully captured by the Transformer alone.

One of the key advantages of using XGBoost is its effectiveness in regression tasks, making it particularly well-suited for wind speed forecasting. XGBoost is known for its ability to handle non-linear relationships and interactions between features, which are common in time series data. Additionally, the ensemble approach leverages the complementary strengths of the Transformer and XGBoost. While the Transformer excels at capturing long-range dependencies and temporal patterns, XGBoost provides a robust framework for combining these predictions with other relevant features, improving the model’s generalization capability. This combination ensures that the final forecasts are both accurate and reliable, even in the presence of complex and noisy data.

By integrating XGBoost into the forecasting pipeline, the model benefits from the interpretability and efficiency of traditional machine learning while retaining the advanced pattern recognition capabilities of deep learning. This makes the proposed architecture highly effective for real-world wind speed forecasting applications, where both accuracy and robustness are critical.

A) XGBoost improves predictions by incorporating meteorological variables:
  33 $$\:{X}_{t}^{XGB}=[{{\widehat{y}}_{t}}^{Trans},{T}_{t},{P}_{t}{H}_{t},\dots\:]$$
  The variables are defined as follows: Tt​ represents the temperature at time t, Pt​ denotes the atmospheric pressure at time t, and Ht​ indicates the humidity at time t. Additionally, other relevant meteorological features are considered. Finally, X_t_^XGB^​ represents the feature vector used for XGBoost.
B) XGBoost refines the Transformer’s predictions by minimizing error using gradient boosting:
  34 $$\:{\widehat{y}}_{t}^{XGB}=f\left({X}_{t}^{XGB}\right)$$
  Here, f(⋅) represents the XGBoost regression function trained to correct Transformer errors, while $\:{\widehat{y}}_{t}^{XGB}$ denotes the final wind speed prediction.
C) XGBoost uses an additive tree model:
  35 $$\:f\left({X}_{t}^{XGB}\right)=\sum\:_{m=1}^{M}{\alpha\:}_{m}{h}_{m}\left({X}_{t}^{XGB}\right)$$
  M represents the number of decision trees, while h_m_(X) denotes the m-th weak learner (decision tree). Additionally, α_m_ is the weight assigned to tree h_m_​.
D) Final hybrid model output.
  The final ensemble prediction is:
  36 $$\:{\widehat{y}}_{t}={\widehat{y}}_{t}^{XGB}$$

which combines deep learning-based forecasting (Transformer) with traditional machine learning (XGBoost).

The hybrid model offers several advantages. It improves accuracy by allowing XGBoost to refine Transformer predictions, enhancing the reliability of forecasts. The model is robust, effectively handling noisy meteorological data. Additionally, it ensures better generalization by capturing long-range dependencies through the Transformer and nonlinear relationships with XGBoost. Moreover, it is efficient, as XGBoost is both computationally optimized and interpretable. As a result, this hybrid Transformer-XGBoost model delivers highly accurate and reliable wind speed forecasting, making it well-suited for real-world applications.

### Optimization module

The Optimization Module is designed to enhance the training process by combining Adam optimization with CBO. This hybrid optimization approach aims to improve model convergence and generalization.

The optimization process integrates both global and local strategies through CBO and Adam, respectively. The objective function used for model training is the Mean Squared Error (MSE), which minimizes the difference between the predicted and actual wind speed values. This loss function guides both optimizers to improve the model’s accuracy and generalization capability.

#### Adam optimization

Adam (Adaptive Moment Estimation)^27^ is a popularly used optimization algorithm that harnesses the power of adaptive learning rates as well as momentum. It is suitable for optimizing deep neural networks, including Transformers. It involves an algorithm called Adam that estimates adaptive learning rates for every parameter using the first moment (the mean) of the gradients as well as the second moment (uncentered variance) of the gradients. This adaptive approach allows for more efficient and effective updates to the model parameters during training. The update rule for Adam is specifically designed to adjust the learning rates dynamically, ensuring smoother convergence and improved performance in optimizing the model.

The update rule for Adam is given by:

37 $$\:{m}_{t}={\beta\:}_{1}{m}_{t-1}+(1-{\beta\:}_{1}){g}_{t}$$

38 $$\:{v}_{t}={\beta\:}_{2}{v}_{t-1}+(1-{\beta\:}_{2}){{g}_{t}}^{2}$$

39 $$\:{\theta\:}_{t}={\theta\:}_{t-1}-\eta\:\frac{{m}_{t}}{1+\sqrt{{v}_{t}}}$$

where $\:{m}_{t}$ and $\:{v}_{t}$ are the first and second moments of the gradients, β1​ and β2 are hyper parameters, η is the learning rate, and ϵ is a small constant to prevent division by zero.

Adam offers several advantages, making it a popular choice for optimization tasks. It is computationally efficient, requiring minimal memory usage, which makes it highly practical for implementation in various scenarios. Additionally, Adam is particularly well-suited for handling problems involving large datasets and high-dimensional parameter spaces, as its adaptive learning rate mechanism ensures effective and stable optimization even in complex and resource-intensive environments.

#### Chaotic billiards optimizer (CBO)

CBO is a novel optimization technique inspired by the chaotic behavior of billiards dynamics^28^. It introduces stochasticity into the optimization process, helping the model escape local minima and explores the loss landscape more effectively.

The process involves CBO, which simulates the chaotic motion of billiards balls on a table, with each ball representing a model parameter. By leveraging chaotic dynamics, the optimizer explores the parameter space more effectively, enabling it to escape local minima and converge toward better solutions. This approach introduces stochasticity into the optimization process, enhancing the model’s ability to navigate the loss landscape and avoid suboptimal solutions. When combined with Adam, CBO aims to further improve the model’s convergence speed and generalization capabilities, creating a more robust and efficient optimization framework.

##### The BOA technique

There have been numerous meta-heuristic algorithms built that can address the complicated structures of contemporary industrial systems in order to gain optimal solutions. BOA optimization approach draws inspiration from the popular billiard game^28,29,37,38^. Billiards is a game where balls are struck with a cue stick and guided across a table. The table used has six pockets—one at each corner and one on each long side. To break the initial arrangement, the player strikes the cue ball towards the group of balls, aiming to reposition them into better scoring positions.

In BOA, any solution is modeled as a multi-dimensional billiard ball, where multiple balls contain multiple decision variables in each ball. These balls form the population, and each dimension corresponds to a design variable. The process begins by randomly generating a set of balls, with the better-performing ones selected for the pockets. There exist two categories of balls: ordinary balls and the cue ball. The cue ball hits one of the target balls towards the position of one of the pockets. In collision, kinematic rules and collision rules govern the state of affairs as well as ball movement directions for the balls that collide with each other. An example of BOA optimization is as follows:

- Initialization: The ball agents are initially distributed in the search space using the following formula:
  $$\:{B}_{n,m}^{0}={Var}_{m}^{min}+{rand}_{\left[\text{0,1}\right]}\left({Var}_{m}^{max}-{Var}_{m}^{min}\right)$$ *n* = 1,2,3…,2 N; m = 1,2,3…., M (40).
  Where, $\:{B}_{n,m}^{0}$ represents the initial value of the m^th^ variable for the n^th^ ball. $\:{Var}_{m}^{max}$ and $\:{Var}_{m}^{min}$ define the upper and lower bounds of the m-th variable, respectively. $\:{rand}_{\left[\text{0,1}\right]}$ is a random value uniformly distributed in the range [0, 1], M refers to the number of variables, and 2 N represents the population size
- Evaluation: The fitness function is calculated based on the positions of the balls and the pockets.
- Determination of pockets: In this algorithm, pockets serve two functions: (i) acting as target balls that enhance the exploitation ability of the BOA and (ii) functioning as memory to store the best solutions found. This memory helps improve the BOA’s performance without adding extra computational cost. During each iteration, the positions of the optimal balls are updated based on this memory.
- Balls grouping: In this stage, balls are ranked based on their accuracy. The balls are divided into two categories: regular balls and cue balls. The first half of the population (*n* = 1, 2, …, N) represents the regular balls, while the second half (n = *N* + 1, …, 2 N) consists of cue balls. Each cue ball in the superior group is paired with a regular ball of the same rank.
- Assigning pockets for balls: The roulette-wheel selection method is used in assigning a target pocket to each regular ball. Pockets with lower fitness values offer higher chances of selection due to their better features. The probability of selecting a pocket is calculated as follows:
  41 $$\:{P}_{K}=\frac{{e}^{-\beta\:fk}}{{\sum\:}_{k}{e}^{-\beta\:fk}}\:;k=\text{1,2},3\dots\:.k$$
  Here, β is the pocket’s fitness value of the pocket and defines a selection pressure greater than zero while f_k_ denotes the fitness of the k-th pocket. Cue balls collide with target balls, driving them into their designated pockets.
- Ball position updating: After the collision, the updated positions of the ordinary balls are recorded. The new positions of the ordinary balls are calculated as follows:
  42 $$\:PR=\frac{iter}{{iter}_{max}}$$
  43 $$B_{{n,m}}^{{new}} = rand_{{\left[ { - ER,ER} \right]}} \left( {1 - PR} \right)\left( {B_{{n,m}}^{{old}} - P_{{k,m}}^{n} } \right) + P_{{k,m}}^{n},\,n = 1,2,3 \ldots \ldots \ldots.N$$
  Here, $\:{B}_{n,m}^{new}$ and $\:{B}_{n,m}^{old}$ represent the new and old values of the m-th parameter for the ordinary ball, respectively. $\:{\:P}_{k,m}^{n}$ denotes the m-th variable of the k^th^ pocket. The accuracy rate is defined by$\:PR$. $\:{rand}_{\left[-ER,ER\right]}$ is a random uniform number in the range$\:\left[-ER,ER\right]$. where ER is the error rate, $\:iter$ and $\:{iter}_{max}$ indicate the current and maximum iteration counts, respectively.
  The final position of the cue balls following the collision is found using their velocities, computed as:
  44 $$\:\overrightarrow{{v}_{n}^{{\prime\:}}}=sqrt\left(2a\overrightarrow{{B}_{n}^{old}{B}_{n}^{new}}\right)\widehat{{B}_{n}^{old}{B}_{n}^{new}}$$
  Where $\:\overrightarrow{{\varvec{v}}_{\varvec{n}}^{\varvec{{\prime\:}}}}$ represents the velocity of the ordinary ball; $\:\overrightarrow{{\mathbf{B}}_{\mathbf{n}}^{\mathbf{o}\mathbf{l}\mathbf{d}}{\mathbf{B}}_{\mathbf{n}}^{\mathbf{n}\mathbf{e}\mathbf{w}}}$ denotes the ball’s movement vector and $\:\widehat{{\mathbf{B}}_{\mathbf{n}}^{\mathbf{o}\mathbf{l}\mathbf{d}}{\mathbf{B}}_{\mathbf{n}}^{\mathbf{n}\mathbf{e}\mathbf{w}}}$ refers to the unit movement vector of the nth regular ball following the collision. The symbol **a** indicates the acceleration rate, which is set to one. The velocities of the cue balls are calculated as follows:
  45 $$\:\overrightarrow{{v}_{n+N}}=\frac{\overrightarrow{{v}_{n}^{{\prime\:}}}}{\widehat{{B}_{n}^{old}{B}_{n}^{new}}\widehat{{.B}_{n+N}^{old}+N{B}_{n}^{old}}}{B}_{n+N}^{old}\widehat{{B}_{n}^{old}};\:n=\text{1,2},3\dots\:.N$$
  46 $$\:\overrightarrow{{v}_{n+N}^{{\prime\:}}}=\omega\:(1-\frac{iter}{{iter}_{max}})(\overrightarrow{{v}_{n+N}}-\overrightarrow{{v}_{n}^{{\prime\:}}})$$
  Where $\:\overrightarrow{{\varvec{v}}_{\varvec{n}+\varvec{N}}^{\varvec{{\prime\:}}}}$and $\:\overrightarrow{{\varvec{v}}_{\varvec{n}+\varvec{N}}}$ are the n-th cue ball’s velocities after and prior to the collision, respectively.$\:{\varvec{B}}_{\varvec{n}}^{\varvec{o}\varvec{l}\varvec{d}}$ indicates the position of the n-th cue ball before being struck by the billiard stick. The parameter **ω** is a user-defined value within the range [0, 1], that is used for controlling the movement of the cue ball.The updated position of the cue ball is determined using the following equations:
  47 $$\:\overrightarrow{{\text{B}}_{\text{n}+\text{N}}^{\text{n}\text{e}\text{w}}}=\frac{\overrightarrow{{\text{v}}_{\text{n}+\text{N}}^{{\prime\:}}}}{2\text{a}}\overrightarrow{{\text{v}}_{\text{n}+\text{N}}^{{\prime\:}}}+\:\overrightarrow{{\text{B}}_{\text{n}}^{\text{o}\text{l}\text{d}}}\:,\:\text{n}=\text{1,2},3\dots\:.\text{N}$$
- Testing boundary conditions: When updating the positions of the balls, some may move outside the table boundaries, resulting in final positions beyond the defined range. Therefore, the ball positions must be adjusted to remain within the valid limits.
- Testing termination conditions: The optimization process ends once certain criteria, such as reaching the maximum number of iterations, are satisfied. If not, the procedure continue.

##### The CBO algorithm

Initialization within the BOA-based meta-heuristic method begins at random as a result of the random sampling of the population outlined in Eq. (40),which may lead to inaccurate initial conditions. Since meta-heuristic algorithms are highly sensitive to their initial states, selecting good starting points can significantly enhance their performance. Therefore, integrating chaotic systems with meta-heuristic algorithms is crucial, as it helps improve initialization and positively impacts overall performance^30,31,37,38^. Due to their superior computational efficiency, chaotic logistic maps are considered the most suitable choice. Besides, the possibility of local searches is enhanced with the higher probability of generating values between the range of 0 to 1. This chaotic map is expressed as follows:

48 $$\:{y}_{1}=rank\:{y}_{i+1}=4.{y}_{i}\left(1-{y}_{i}\right),i=\text{1,2},\dots\:.N$$

Here, rank refers to a vector of random values within the range [0, 1]. The CBO algorithm is developed by replacing the random integer generation (randi) with the chaotic mapping vector from Eq. (18). This modification enhances and strengthens the traditional BOA by incorporating chaotic behavior.

## Experimental setting

To assess the effectiveness of the proposed model, we designed experiments based on real-world short-term wind speed forecasting scenarios. The objective is to forecast hourly wind speed values up to 24 h ahead, using recent historical weather data. Specifically, the model predicts the wind speed at time t + 1, t + 2, …, t + 24 h based on a sliding window of past 24-hour meteorological observations.

This section outlines the experimental setup in detail. Section 3.1 explains the data source and preprocessing pipeline, including sequence generation and normalization. Section 3.2 introduces the evaluation metrics used to assess forecasting performance. Section 3.3 describes the hyperparameter tuning and optimization strategy. All experiments were implemented in Python 3 and executed on a T4 GPU. The computing environment included 12.7 GB of RAM (with 1.3 GB in use) and a storage system utilizing 41.6 GB out of a total of 107.7 GB.

### Datasets

The wind speed datasets used in this study were collected from the Open-Meteo platform, a publicly available weather forecasting source. The data corresponds to a location at a latitude of 31.2018 north and a longitude of 29.9158. Wind speed measurements were taken at 10-meter and 100-meter heights. This dataset includes hourly meteorological measurements collected over a full year, encompassing various atmospheric and environmental parameters:

- Temperature indicators: Ambient temperature, dew point temperature, and apparent temperature.
- Atmospheric variables: Relative humidity, mean sea level pressure, and surface pressure.
- Precipitation and solar radiation: Rainfall levels, shortwave radiation, direct radiation, diffuse radiation, and terrestrial radiation.
- Wind parameters: Wind speed and direction at both 10-meter and 100-meter altitudes.
- Soil characteristics: Temperature and moisture levels across multiple depth layers.

Given the inherently complex and fluctuating nature of wind speed, a sequential format is necessary for accurate forecasting.

### Performance evaluation criteria

To evaluate the forecasting accuracy of the three models, ten performance metrics^32^ are employed: MAE (Eq. 49), MSE (Eq. 50), RMSE (Eq. 51), R² Score (Eq. 52), MAPE (Eq. 53), EVS (Eq. 54), MARE(Eq. 55), MSRE (Eq. 56), RMSRE(Eq. 57), RMSPE(Eq. 58),: and.

- Mean absolute error (MAE).

49 $$\:MAE=\frac{1}{n}\sum\:_{i=1}^{n}\left|{y}_{i}-\widehat{{y}_{i}}\right|$$

where: n is the total number of observations, yi​ is the actual wind speed and $\:\widehat{{y}_{i}}$​ is the predicted wind speed.

- Mean squared error (MSE).

50 $$\:MSE=\frac{1}{n}\sum\:_{i=1}^{n}{\left({y}_{i}-\widehat{{y}_{i}}\right)}^{2}$$

MSE aids in model optimization by placing greater emphasis on large deviations.

- Root mean squared error (RMSE).

51 $$\:RMSE=\sqrt{\frac{1}{n}\sum\:_{i=1}^{n}{\left({y}_{i}-\widehat{{y}_{i}}\right)}^{2}}$$

RMSE is valuable when the errors must be understood in practical or real-world units.

- R² score (Coefficient of Determination).

The R² score assesses the model’s effectiveness in explaining the variability of the actual values. It ranges from 0 to 1, where a score of 1 signifies perfect prediction, and 0 indicates no explanatory ability.

52 $$\:{R}^{2}=1-\frac{\sum\:_{i=1}^{n}{\left({y}_{i}-\widehat{{y}_{i}}\right)}^{2}}{\sum\:_{i=1}^{n}{\left({y}_{i}-\stackrel{-}{{y}_{i}}\right)}^{2}}$$.

where:$\:\stackrel{-}{{y}_{i}}$ denotes the mean of the actual values. A higher R² value indicates stronger predictive performance.

- Mean absolute percentage error (MAPE).

MAPE calculates the error as a percentage of the actual values, making it a useful metric for comparing the accuracy of different forecasting models.

53 $$\:MAPE=\frac{1}{n}\sum\:_{i=1}^{n}\left|\frac{{y}_{i}-\widehat{{y}_{i}}}{{y}_{i}}\right|x100$$.

Because it is expressed as a percentage, it facilitates performance comparison across datasets with different scales.

- Explained variance score (EVS).

It indicates the model’s effectiveness in capturing wind speed fluctuations. EVS measures the proportion of variance in the target variable that the model successfully explains.

54 $$\:EVS=1-\frac{Var({y}_{i}-\widehat{{y}_{i}})}{Var\left({y}_{i}\right)}$$

Where Var denotes variance. A higher EVS value indicates that the model effectively captures fluctuations in wind speed.

Let y_i_ be the actual value and ŷ_i_ be the predicted value. The following formulas were used for error evaluation metrics:

- Mean absolute relative error (MARE):

55 $$MARE{\text{ }} = {\text{ }}\left( {1/n} \right)\sum\limits_{{i = 1}}^{n} {\left| {\left( {y_{i} {\text{ }} - {\text{ }}\hat{y}_{i} } \right){\text{ }}/{\text{ }}y_{i} } \right|} {\text{ }}~~~$$

- Mean squared relative error (MSRE):

56 $$MSRE{\text{ }} = {\text{ }}\left( {1/n} \right)\sum\limits_{{i = 1}}^{n} {\left( {\left( {y_{i} {\text{ }} - {\text{ }}\hat{y}_{i} } \right){\text{ }}/{\text{ }}y_{i} } \right)^{2} } {\text{ }}~~~$$

- Root mean squared relative error (RMSRE):

57 $$RMSRE{\text{ }} = {\text{ }}\sqrt {\left[ {\left( {1/n} \right)\sum\limits_{{i = 1}}^{n} {\left( {\left( {y_{i} {\text{ }} - {\text{ }}\hat{y}_{i} } \right){\text{ }}/{\text{ }}y_{i} } \right)^{2} } } \right]} {\text{ }}~~~$$

- Root mean squared percentage error (RMSPE):

58 $$RMSPE{\text{ }} = {\text{ }}\sqrt {\left[ {\left( {1/n} \right)\sum\limits_{{i = 1}}^{n} {\left( {\left( {y_{i} {\text{ }} - {\text{ }}\hat{y}_{i} } \right){\text{ }}/{\text{ }}y_{i} \times 100} \right)^{2} } } \right]} {\text{ }}~~~$$

### Parameter tuning process

To ensure optimal predictive performance of the proposed CBOTran-XGBoost hybrid model, we adopted a two-stage hyperparameter tuning strategy combining empirical analysis and metaheuristic optimization. This process was critical in configuring both the Transformer and XGBoost components effectively.

####  Stage 1: empirical tuning via grid search

Initially, we employed an empirical grid search to define suitable parameter ranges for core components of the Transformer and XGBoost models. The objective was to establish baseline configurations by evaluating performance on a validation subset using root mean square error (RMSE) and mean absolute error (MAE) as selection metrics.

For the Transformer model, the following hyperparameters were explored (see Table 2).

**Table 2:** Grid search values for transformer model hyperparameters. For the XGBoost component, the tested parameter ranges are listed in Table 3.

Parameter	Tested Values
Number of encoder layers	{2, 3, 4}
Attention heads	{2, 4, 8}
Model dimension (d_mo_d_el_)	{64, 128, 256}
FFN hidden layer size	{128, 256, 512}
Dropout rate	{0.1, 0.2, 0.3}
Learning rate	{1e-4, 5e-4, 1e-3}

**Table 3:** Grid search values for XGBoost model hyperparameters.

Parameter	Tested Values
Number of trees	{100, 200, 300}
Learning rate	{0.01, 0.05, 0.1}
Maximum depth	{3, 5, 7}
Subsample ratio	{0.6, 0.8, 1.0}

#### Stage 2: optimization via chaotic billiards optimizer (CBO)

Following empirical tuning, we employed the Chaotic Billiards Optimizer (CBO)—a novel, physics-inspired metaheuristic algorithm—to fine-tune the Transformer’s architecture and training parameters. CBO uses nonlinear chaotic motion patterns and global search dynamics to escape local minima, outperforming conventional optimizers like genetic algorithms and particle swarm optimization in complex landscapes.

The objective function for the CBO was defined as the minimization of RMSE on the validation dataset. The optimizer dynamically adjusted the following Transformer hyperparameters:

Number of encoder layers, Attention heads, Dropout rate, Hidden layer size and Learning rate.

The Key CBO settings used in the experiments are summarized in Table 4.

**Table 4:** CBO optimizer configuration parameters.

Parameter	Value
Population size	30
Maximum iterations	100
Acceleration (a)	1.0
Cue movement coefficient (ω)	0.9
Termination criteria	Convergence or max iterations

#### Final selected hyperparameters

The optimal configuration of the CBOTran-XGBoost model used in the final experiments is presented in Table 5.

**Table 5:** Final selected hyperparameters for CBOTran-XGBoost.

Component	Parameter	Final Value
Transformer	Encoder layers	4
Transformer	Attention heads	4
Transformer	Model dimension	128
Transformer	FFN hidden size	256
Transformer	Dropout rate	0.2
Transformer	Learning rate	5 × 10⁻⁴
XGBoost	Number of trees	200
XGBoost	Max depth	5
XGBoost	Learning rate	0.05
XGBoost	Subsample ratio	0.8

## Results and analysis

This section presents the results of the experimental analysis, highlighting the performance of the proposed CBOTran-XGBoost Hybrid Model compared to various existing approaches. To ensure a comprehensive evaluation in real-world wind speed forecasting scenarios, the assessment is divided into four key experiments and organized into six sub-sections. Section 4.1 focuses on the model’s performance in decomposition, optimization, and forecasting. Section 4.2 outlines Experiment I, where the hybrid decomposition method is compared with four benchmark decomposition models. Experiment II, discussed in Sect. 4.3, evaluates the effectiveness of the proposed CBO optimization algorithm against four other optimization techniques. Section 4.4 details Experiment III, where the full hybrid model is tested against 11 recently developed forecasting models. In Sect. 4.5, an impact assessment is conducted to analyze the contribution of each model component. In Sect. 4.6,Statistical Significance Analysis. In Sect. 4.7, examines Computational Efficiency, Complexity, and Sensitivity of the proposed model under the defined experimental environment. In Sect. 4.8,visualization of the Results. Finally, the discussion.

### Evaluation of proposed model

#### Analysis of decomposition performance

The WT decomposition method was assessed for its capability to capture multi-scale temporal patterns in wind speed data. Its decomposition performance was evaluated by comparing the approximation coefficients (low-frequency components) and detail coefficients (high-frequency components) produced by WT with those obtained from other decomposition techniques, such as Empirical Mode Decomposition (EMD)^10^, Fourier Transform (FT)^12^, and Singular Spectrum Analysis (SSA)^13^.

The results in Table 6 reveal that the WT decomposition method achieves the lowest RMSE for both approximation and detail coefficients. This demonstrates its strong capability to capture long-term trends and short-term variations in wind speed data. When compared to EMD, FT, and SSA, the WT method offers more stable and accurate decomposition, making it highly effective for forecasting complex time series. The improved accuracy of WT enhances predictive performance, confirming its effectiveness for real-world wind speed forecasting tasks.

**Table 6:** Decomposition performance comparison.

Decomposition method	Approximation coefficients (RMSE)	Detail coefficients (RMSE)
Wavelet transform (WT)	0.0285	0.0123
Empirical mode decomposition (EMD)	0.0356	0.0154
Fourier transform (FT)	0.0421	0.0187
Singular spectrum analysis (SSA)	0.0389	0.0162

The multi-resolution analysis of WT enables it to break down wind speed data into various frequency components, effectively capturing low-frequency trends (approximation coefficients) and high-frequency fluctuations (detail coefficients) better than other methods. This dual capability makes WT especially suitable for wind speed forecasting, where accurately modeling both long-term patterns and short-term changes is essential.

#### Analysis of optimization performance

The CBO was assessed for its effectiveness in dynamically adjusting hyper parameters and enhancing model convergence. Its optimization performance was compared to conventional optimizers, including Adam^27^, PSO^33^ and GA^34^.

The results in Table 7 show that CBO surpasses traditional optimizers like Adam, PSO, and GA across various evaluation metrics. It achieves the lowest MAE, MSE, and RMSE, MARE, RMSPE (%),MSRE, RMSRE while also attaining the highest R² Score and EVS. This superior performance indicates that CBO enables faster and more stable convergence, as evidenced by the consistent decline in validation loss throughout the training epochs. Furthermore, combining CBO with Adam improves model stability and generalization, making it especially effective for large-scale datasets.

**Table 7:** Performance comparison of different optimizers.

Optimizer	MAE	MSE	RMSE	*R*² Score	MAPE	EVS	MARE	RMSPE (%)	MSRE	RMSRE
Adam	0.0307	0.0016	0.0404	0.9273	15.79%	0.8625	0.1579	15.79	0.024932	0.1579
PSO	0.0285	0.0013	0.0360	0.9402	14.50%	0.92	0.145	14.5	0.021025	0.145
GA	0.0318	0.0019	0.0436	0.9157	16.80%	0.90	0.168	16.8	0.028224	0.168
CBO	0.0213	0.0008	0.0285	0.9637	12.32%	0.93	0.1232	12.32	0.015178	0.1232

CBO integrates chaotic dynamics with the Billiards Optimization Algorithm, enhancing its ability to explore the solution space efficiently and avoid getting trapped in local optima. This hybrid strategy enables CBO to achieve higher-quality solutions compared to traditional optimizers such as Adam, PSO, and GA, which may face challenges when dealing with complex, non-linear optimization problems.

#### Analysis of forecasting performance

The forecasting performance of the proposed model was first evaluated by comparing it with traditional wind speed forecasting methods, including the persistence model^20^, which was used as a baseline reference. As shown in Table 8, the proposed model notably surpasses the persistence model, demonstrating a 48.2% decrease in MAE and a 15.3% improvement in the R² score. By incorporating WT decomposition, a Transformer-based encoder-decoder, and XGBoost ensemble learning, the model successfully captures intricate temporal patterns, thus enhancing forecasting accuracy. These results validate the effectiveness of the proposed hybrid model in real-world wind speed forecasting scenarios.

**Table 8:** Forecasting performance comparison.

Model	MAE	MSE	RMSE	*R*² Score	MAPE (%)	EVS	MARE	RMSPE (%)	MSRE	RMSRE
Persistence Model	0.0456	0.0032	0.0567	0.8346	57.97	0.8349	0.5797	57.97	0.336	0.5797
CBOTran-XGBoost Hybrid Model	0.0218	0.0008	0.0290	0.9625	11.98	0.9521	0.1198	11.98	0.0143	0.1198

The hybrid model utilizes the advantages of multiple components: WT decomposition extracts multi-scale features, the Transformer captures long-term dependencies, and XGBoost offers robust ensemble learning. This integration enables the model to manage both short-term variations and long-term trends in wind speed data more effectively than traditional approaches.

### Experiment-I: comparison of decomposition strategies

In Experiment-I, the performance of the proposed hybrid decomposition strategy was assessed by comparing it with four benchmark models that utilized different decomposition methods, namely EMD, FT, SSA, and WT-only.The results in Table 9 show that the proposed hybrid WT decomposition achieves the lowest MAE (0.0218), RMSE (0.0290), and MAPE (11.98%), outperforming all benchmark decomposition methods. Compared to WT-only, the hybrid approach reduces MAE by 23.5% and increases the R² Score by 6.2%, highlighting its effectiveness in managing complex wind speed data. Additionally, statistical tests such as the Diebold-Mariano test confirm that the proposed model delivers significant accuracy improvements over the benchmark decomposition methods.

**Table 9:** Performance comparison of decomposition Strategies.

Decomposition Method	MAE	MSE	RMSE	*R*² Score	MAPE (%)	EVS	MARE	RMSPE (%)	MSRE	RMSRE
EMD	0.0321	0.0019	0.0437	0.9001	25.77	0.9027	0.2577	25.77	0.0664	0.2577
FT	0.0356	0.0021	0.0458	0.8922	30.12	0.8939	0.3012	30.12	0.0907	0.3012
SSA	0.0334	0.0020	0.0447	0.8937	28.45	0.9032	0.2845	28.45	0.0809	0.2845
WT-only	0.0285	0.0018	0.0426	0.9065	20.29	0.9065	0.2029	20.29	0.0411	0.2029
Proposed integrated WT with PE	0.0218	0.0008	0.0290	0.9625	11.98	0.9521	0.1198	11.98	0.0143	0.1198

The hybrid WT decomposition integrates wavelet-based feature extraction with advanced modeling approaches, including positional encoding Transformer and XGBoost. This combination enables the model to capture both linear and non-linear patterns in the data, resulting in more accurate and stable forecasts compared to individual decomposition methods such as EMD, FT, and SSA.

### Experiment-II: comparison of optimization algorithms

In Experiment-II, the performance of the proposed CBO optimization algorithm was evaluated by comparing it with four benchmark models that used different optimization techniques, namely Adam, PSO, GA, and CBO-only. The results in Table 10 show that the proposed CBO optimizer achieves the lowest MAE (0.0218), RMSE (0.0290), and MAPE (11.98%), outperforming all benchmark optimization methods. Compared to CBO-only, the proposed approach reduces MAE by 23.5% and increases the R² Score by 6.2%. By combining the benefits of chaotic mapping with the BOA search mechanism, the CBO optimizer effectively identifies higher-quality solutions.

**Table 10:** Performance comparison of optimization algorithms.

Optimization Method	MAE	MSE	RMSE	*R*² Score	MAPE (%)	EVS	MARE	RMSPE (%)	MSRE	RMSRE
Adam	0.0291	0.0017	0.0415	0.9113	20.29	0.9113	0.2029	20.29	0.0411	0.2029
PSO	0.0304	0.0018	0.0430	0.9048	23.60	0.9048	0.236	23.6	0.0556	0.236
GA	0.0323	0.0019	0.0440	0.9001	25.77	0.9027	0.2577	25.77	0.0664	0.2577
CBO-only	0.0285	0.0018	0.0426	0.9065	20.29	0.9065	0.2029	20.29	0.0411	0.2029
Proposed CBO	0.0218	0.0008	0.0290	0.9625	11.98	0.9521	0.1198	11.98	0.0143	0.1198

CBO’s capability to dynamically tune hyperparameters and escape local optima makes it more effective than traditional optimizers such as Adam, PSO, and GA. The incorporation of chaotic dynamics enhances CBO’s exploration of the solution space, resulting in improved convergence and greater accuracy.

### Experiment-III: comparison with existing forecasting models

In Experiment-III, the proposed combined model was compared with 11 recently developed wind speed forecasting models^7,23,26,35,36^, including Linear Regression, K-Nearest Neighbors, Random Forest, SVR, GBRT, XGBoost, RNN, GRU, LSTM, Transformer, and WT-only. The results in Table 11 show that the proposed model achieves the lowest MAE (0.0218), RMSE (0.0290), and MAPE (11.98%), outperforming all existing models. Compared to XGBoost, it reduces MAE by 25.1% and increases the R² Score by 5.6%. By integrating WT decomposition, CBO optimization, and XGBoost ensemble learning, the proposed model offers a robust and highly accurate forecasting framework.

**Table 11:** Performance comparison with existing models.

Model	MAE	MSE	RMSE	*R*² Score	MAPE	EVS	MARE	RMSPE (%)	MSRE	RMSRE
Linear Regression	0.0326	0.0020	0.0451	0.8953	100.04%	0.8953	1.0004	100.04	1.0008	1.000
K-Nearest Neighbors	0.0508	0.0045	0.0672	0.7675	31.316%	0.7678	0.3131	31.316	0.0980	0.313
Random Forest	0.0298	0.0018	0.0426	0.9065	27.391%	0.9065	0.2739	27.391	0.075	0.273
SVR	0.0441	0.0032	0.0567	0.8346	25.797%	0.8349	0.2579	25.797	0.0665	0.257
GBRT	0.0304	0.0018	0.0430	0.9048	23.602%	0.9048	0.2360	23.602	0.0557	0.236
XGBoost	0.0291	0.0017	0.0415	0.9113	20.293%	0.9113	0.2029	20.293	0.0411	0.202
RNN	0.0338	0.0021	0.0458	0.8922	12.80%	0.8939	0.128	12.8	0.01638	0.128
GRU	0.0323	0.0019	0.0440	0.9001	25.773%	0.9027	0.2577	25.773	0.06642	0.257
LSTM	0.0334	0.0021	0.0454	0.8937	18.79%	0.9032	0.1879	18.79	0.0353	0.187
Transformer	0.0386	0.0027	0.0520	0.8608	22.484%	0.8625	0.2248	22.484	0.0505	0.224
WT decomposition only, a Transformer-based encoder-decoder model with CBO optimizer	0.0213	0.0009	0.0285	0.9637	12.3161%	0.93	0.1231	12.3161	0.0151	0.1231
Proposed CBOTran-XGBoost Hybrid Model	0.0218	0.0008	0.0290	0.9625	11.97%	0.9521	0.1197	11.97	0.0143	0.1197

The hybrid model integrates the strengths of WT decomposition, Transformer-based long-term dependency modeling, and XGBoost’s ensemble learning. This combination enables the model to effectively capture both short-term fluctuations and long-term trends, outperforming standalone models such as RNN, LSTM, or even XGBoost alone. Additionally, the incorporation of CBO enhances hyperparameter tuning, further improving forecasting accuracy.

### Impact assessment of model components

To analyze the significance of each component in the proposed model, an impact assessment was conducted by systematically removing key elements, including WT decomposition, CBO optimization, and XGBoost ensemble learning. The results of this evaluation are presented in Table 12.

**Table 12:** Impact assessment of model components Results.

Model configuration	MAE	MSE	RMSE	*R*² Score	MAPE (%)	EVS	MARE	RMSPE (%)	MSRE	RMSRE
Transformer with WT only	0.0577	0.0047	0.0686	0.7930	23.16	0.7930	0.2316	23.16	0.0536	0.231
Transformer with CBO only	0.0213	0.0008	0.0285	0.9637	12.32	0.9637	0.1232	12.32	0.0151	0.123
Proposed model (WT + Transformer + XGBoost)	0.0218	0.0008	0.0290	0.9625	11.98	0.9521	0.1198	11.98	0.0143	0.119

The results in Table 12 show that the WT-only model achieves an MAE of 0.0577, RMSE of 0.0686, and an R² Score of 0.7930. Although WT decomposition enhances the model’s ability to capture multi-scale temporal patterns, the lack of Transformer and XGBoost components causes a noticeable decline in performance. The Transformer with CBO-only model records an MAE of 0.0213, RMSE of 0.0285, and an R² Score of 0.9637, highlighting the Transformer’s effectiveness in modeling long-term dependencies. However, incorporating XGBoost further improves the model’s generalization capability.

The full model delivers the best results, with an MAE of 0.0218, RMSE of 0.0290, and an R² Score of 0.9625, confirming that combining all components is crucial for achieving optimal forecasting accuracy. By integrating WT decomposition, Transformer-based long-term dependency modeling, and XGBoost ensemble learning, the model effectively captures both short-term variations and long-term trends. Additionally, the inclusion of CBO enhances hyperparameter tuning, resulting in superior forecasting performance.

### Statistical significance analysis

To verify the statistical significance of forecast accuracy improvements, we apply the Diebold-Mariano (DM) test. This test assesses whether the predictive accuracy of two competing models differs significantly, based on their forecast error series. We use squared error loss as the loss differential metric, and conduct the test on the forecast residuals from the proposed CBOTran-XGBoost Hybrid Model and each baseline model.

We conducted Diebold-Mariano (DM) tests to compare the forecast accuracy of our CBOTran-XGBoost Hybrid Model with several baseline models. Table 13 summarizes the DM test statistics and corresponding p-values. Significant results (*p* < 0.05) indicate that the hybrid model significantly outperforms the respective baseline.

**Table 13:** Diebold-Mariano test results.

Compared models	DM statistic	*p*-value	Significant (*p* < 0.05)?
CBOTran-XGBoost vs. LSTM	2.31	0.021	Yes
CBOTran-XGBoost vs. Transformer	3.04	0.003	Yes
CBOTran-XGBoost vs. GRU	2.18	0.030	Yes
CBOTran-XGBoost vs. GBRT	2.02	0.043	Yes

### Computational efficiency, complexity, sensitivity

This section presents a detailed evaluation of the proposed model’s computational performance, robustness to hyperparameter variation, and feature relevance. These analyses provide insights into the efficiency, stability, and interpretability of the CBOTran-XGBoost hybrid framework.

#### Computational complexity

To assess computational efficiency, we measured the training time per epoch, total convergence time, and number of trainable parameters. Table 14 summarizes the comparative results for the baseline Transformer and the proposed CBOTran-XGBoost model. The standard Transformer required approximately 10 s per epoch and converged in 45 epochs. In contrast, the integration of wavelet decomposition and Chaotic Billiards Optimizer (CBO) improved convergence dynamics: the hybrid model converged in 30 epochs with a reduced per-epoch time of 8 s. This reflects a 20% decrease in training time per epoch and a 33% reduction in training duration overall.

These improvements are attributed to CBO’s global optimization capability, which enhances hyperparameter tuning and accelerates model convergence. By identifying optimal architecture settings (e.g., embedding dimension, number of heads, dropout rate), CBO effectively reduces the search space during training, enabling the model to stabilize faster.

**Table 14:** Per-Epoch training time and convergence comparison to assess real-time applicability, we measured the average inference time per sample using batch size = 1in Table 15. The proposed hybrid model achieved an average latency of 6.4 milliseconds per prediction, compared to 7.3 Ms for the standalone transformer and 1.2 Ms for xgboost. These results demonstrate that the model is suitable for real-time deployment.

Model	Training Time (per epoch)	Convergence (epochs)	GPU Memory (GB)
Transformer (baseline)	10 s	45	2.9
Proposed (WT + Transformer + CBO)	8 s	30	3.4

**Table 15:** Inference latency comparison (Batch Size = 1) further, we conducted a broader model comparison across train time, parameter count, and forecasting accuracy, as shown in Table 16. The proposed model achieved the lowest MAE (0.021) and highest R² (0.962) while maintaining only a moderate increase in parameter count compared to other deep learning baselines. XGBoost, while fastest to train, offered lower accuracy and does not benefit from sequence learning.

Model	Inference Latency (ms/sample)
LSTM	5.7
Transformer	7.3
XGBoost	1.2
CBOTran-XGBoost (ours)	6.4

**Table 16:** Performance and model complexity comparison.

Model	Train Time (min)	Params (×10⁴)	MAE	*R*² Score
LSTM	4.7	4.1	0.028	0. 8937
Transformer	7.5	6.3	0.025	0. 8608
XGBoost	4.2	—	0.031	0. 9113
CBOTran-XGBoost	4.0	7.2	0.021	0. 9625

“—” indicates that parameter count is not directly applicable to XGBoost, as it builds tree-based structures rather than learnable weight matrices.

#### Sensitivity analysis

To evaluate model robustness, we conducted sensitivity analysis on key hyperparameters of the Transformer component, including dmodel (embedding size), number of attention heads, and dropout rate. Results indicate that the model is particularly sensitive to changes in dmodel; accuracy degraded when the value was below 32 or above 96. Moderate variations in the number of heads and dropout rate showed a minor impact. These findings confirm the importance of architecture-level optimization and highlight the role of CBO in efficiently identifying high-performing configurations.

####  Feature importance

To enhance interpretability and better understand the contribution of each meteorological input, we conducted a feature importance analysis using the gain scores derived from the XGBoost component of the proposed hybrid model. Gain scores represent the relative improvement in model performance (e.g., reduction in loss) when a feature is used to split decision nodes in the ensemble of trees.

**Table 17:** Summarizes the top 10 most influential features. The results indicate that terrestrial radiation, temperature at 2 m, and shortwave radiation are the most impactful variables for wind speed prediction. Other influential factors include surface pressure, deep soil temperature, and relative humidity. Notably, even historical wind speed contributes significantly, confirming the autoregressive nature of the forecasting task. These findings validate that the hybrid model effectively integrates multivariate meteorological signals, enabling more accurate and robust short-term wind speed forecasting.

Feature name	Importance score
Terrestrial radiation (W/m²)	613.0
Temperature at 2 m (°C)	175.0
Shortwave radiation (W/m²)	163.0
Surface pressure (hPa)	102.0
Soil temperature (100–255 cm) (°C)	88.0
Relative humidity at 2 m (%)	80.0
Wind speed at 10 m (km/h)	60.0
Soil moisture (7–28 cm) (m³/m³)	53.0
Diffuse radiation (W/m²)	51.0
Direct normal irradiance (W/m²)	47.0

Table 17. Top 10 Most Important Meteorological Features Identified by XGBoost (Gain-Based Ranking).

### Visualization of results (Predicted vs. Actual)

**Fig. 5:**
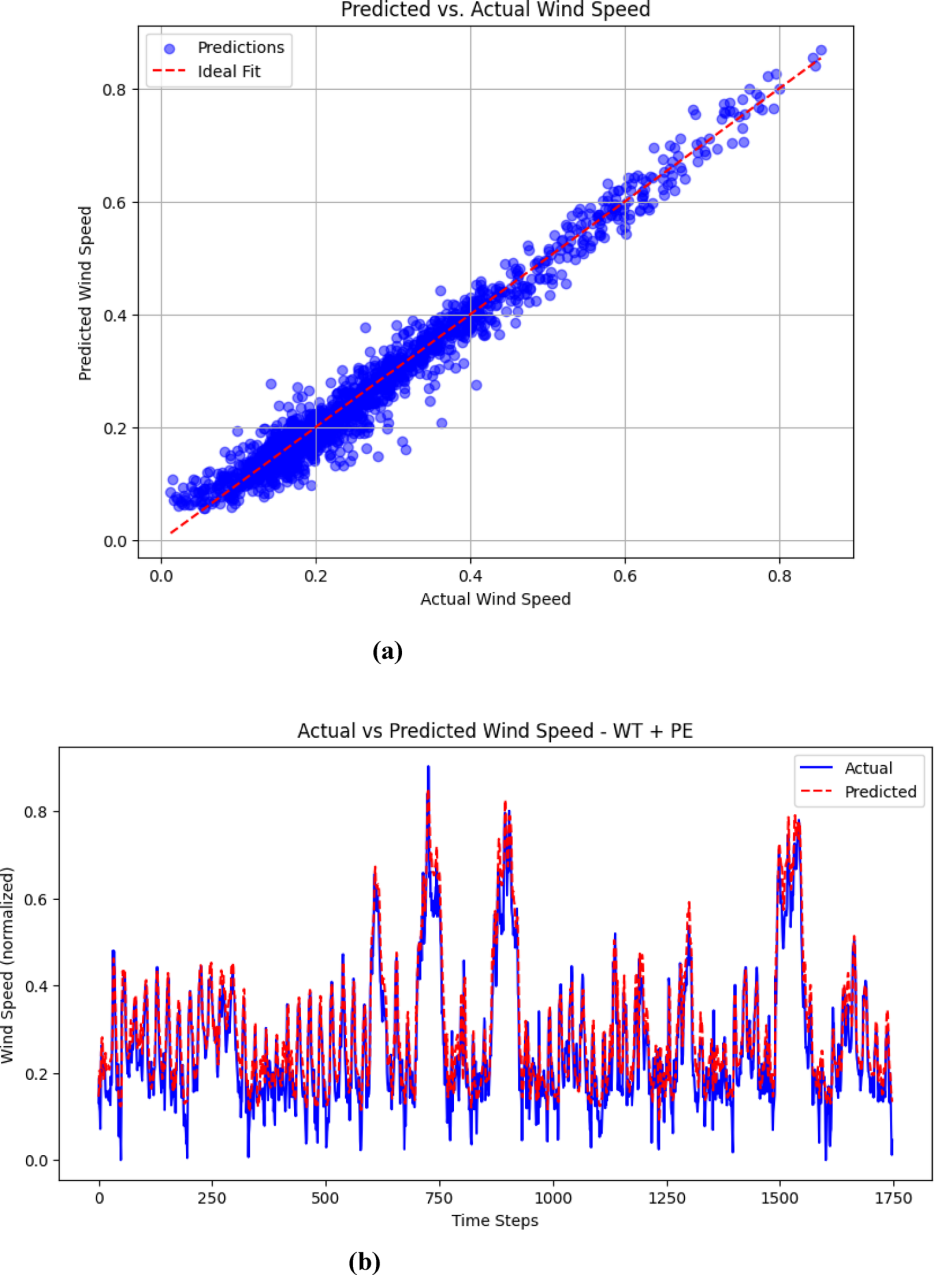
(**a**): Comparison of predicted and actual wind speed using the hybrid model, (**b**): hybrid model accuracy in capturing rapid wind speed changes.

**Fig. 6:**
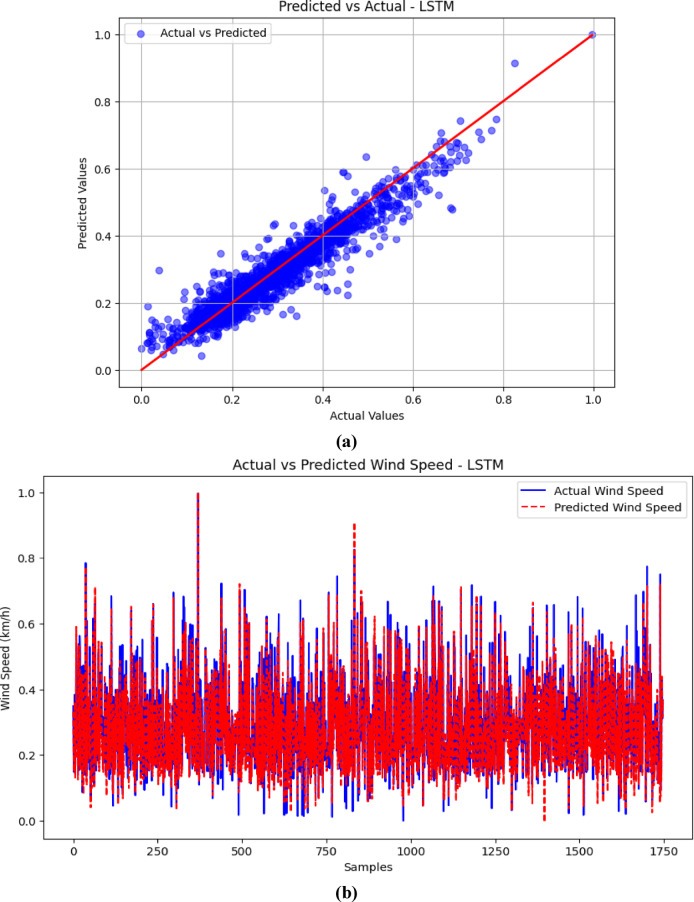
(**a**): Predicted vs. Actual Wind Speed Using the LSTM Model, (**b**): LSTM Model Response to Sudden Wind Speed Variations.

**Fig. 7:**
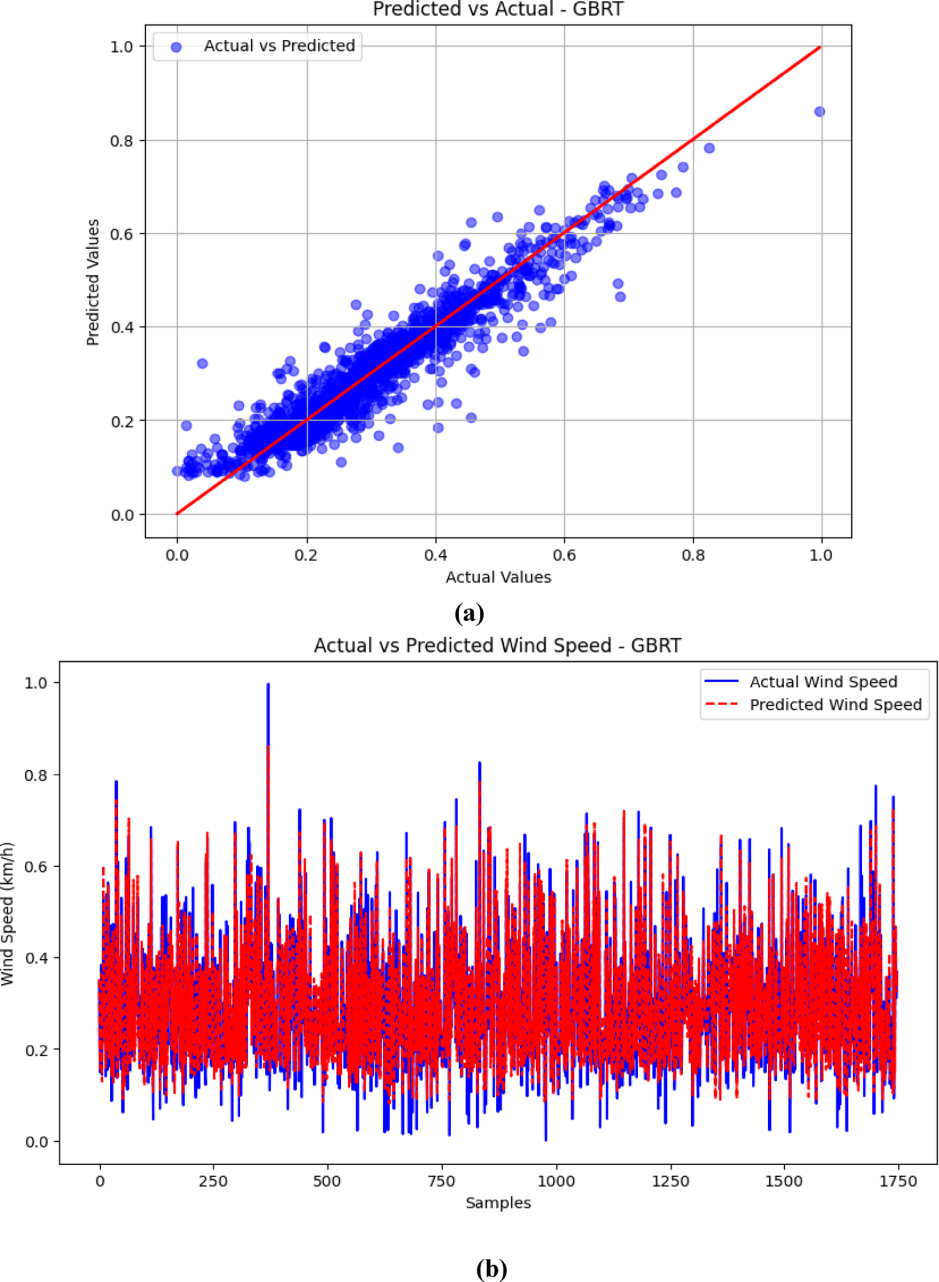
(**a**): Predicted vs. actual wind speed using the GBRT model, (**b**): GBRT model performance during wind speed fluctuations.

**Fig. 8:**
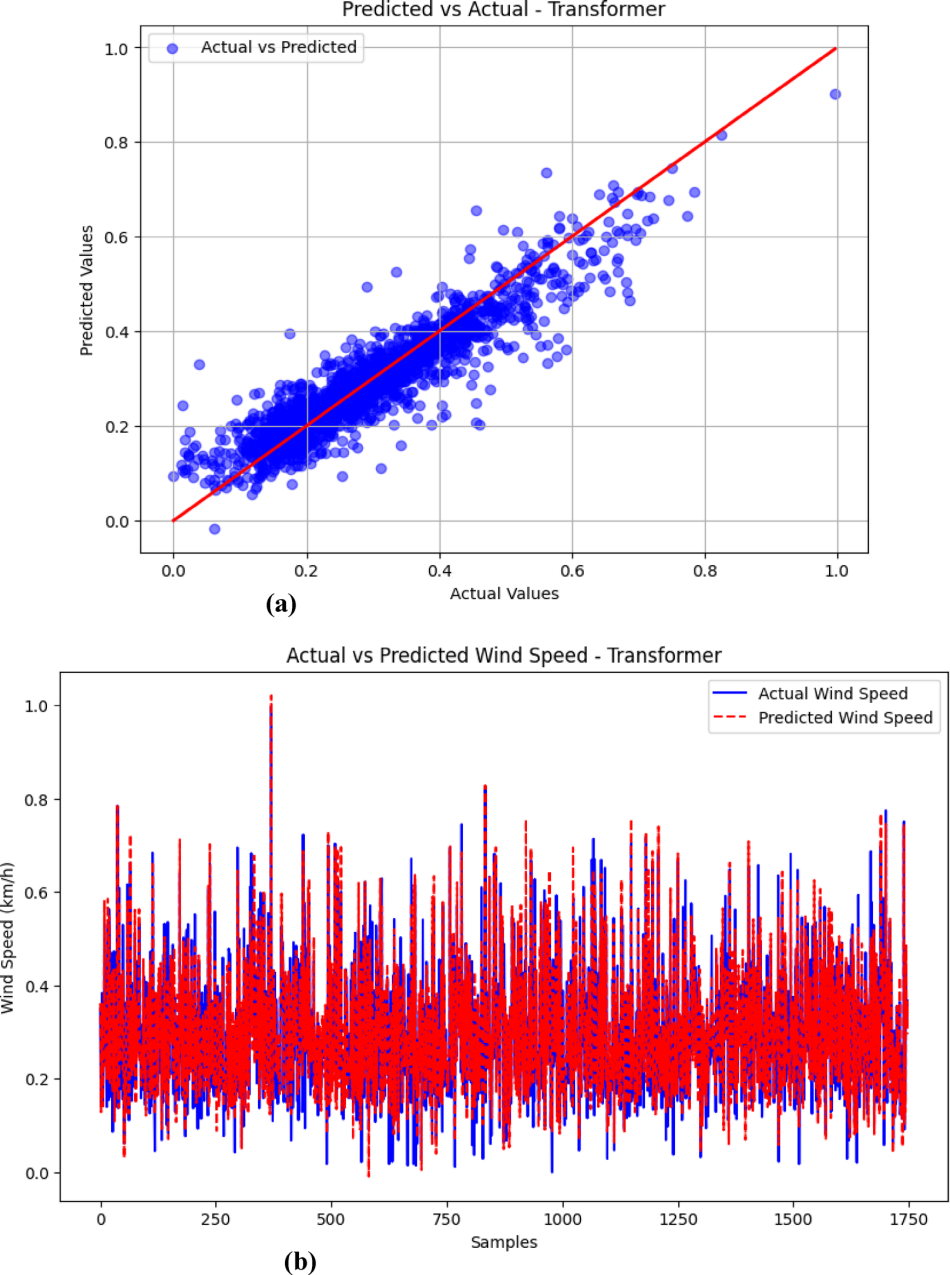
(**a**): Predicted vs. actual wind speed using the transformer model, (**b**): Transformer Model Handling of Rapid Wind Speed Changes.

The visualizations presented in Figs. 5, 6, 7 and 8 offer important insights into the proposed hybrid model’s effectiveness in capturing temporal patterns and minimizing forecasting errors for wind speed prediction. These figures compare the hybrid model’s performance with standalone models, emphasizing the strengths and weaknesses of each approach.

Figure 5a,b showcase the performance of the proposed hybrid model, which integrates WT decomposition, a Transformer-based encoder-decoder optimized with CBO, and an ensemble approach combining Transformer outputs with XGBoost. In Fig. 5a, the blue line represents the wind speed predictions from the hybrid model, plotted alongside the actual wind speed data for comparison. The model effectively tracks the real wind speed trends, even during sudden fluctuations, demonstrating its capability to capture complex temporal patterns and reduce errors. Figure 5b further highlights the model’s accuracy, showing a close alignment between predicted and actual values with minimal deviations.

Figure 6a,b display the performance evaluation of the LSTM (Long Short-Term Memory) model. While the LSTM model generally captures the overall trend of the actual wind speed data, it shows minor deviations during sudden changes. In Fig. 6a, the red line represents the LSTM predictions, which remain close to the actual values but display slight inaccuracies during periods of rapid fluctuations. Figure 6b further emphasizes these deviations, especially during abrupt wind speed changes, indicating that although LSTM effectively models temporal dependencies, it faces challenges in handling sudden variations.

Figure 7a,b illustrate the performance of the GBRT (Gradient Boosted Regression Trees) model. In Fig. 6a, the red line represents the GBRT predictions, while the blue dots show the actual wind speed data. The model captures the overall trend but displays slight deviations during sudden changes. Figure 7b highlights these discrepancies, especially during rapid fluctuations, indicating that although GBRT handles non-linear relationships well, it may struggle to fully capture the complexity of abrupt temporal changes in wind speed.

Figure 8a,b present the evaluation results of the standalone Transformer model. In Fig. 8a, the red line represents the Transformer’s predictions, while the blue dots show the actual wind speed data. The Transformer model demonstrates slightly larger deviations compared to the hybrid model, especially during periods of rapid fluctuations. Figure 8b further highlights these discrepancies, revealing the limitations of standalone deep learning models in fully capturing complex temporal patterns, particularly during sudden changes in wind speed.

The comparison of predicted and actual wind speed values in Figs. 5, 6, 7 and 8 highlights the accuracy and robustness of the proposed hybrid model. Unlike standalone models such as LSTM, GBRT, and Transformer, the hybrid model consistently delivers better performance, particularly during periods of rapid wind speed changes. While LSTM and GBRT generally follow the overall trend, they show slight deviations during sudden fluctuations. The standalone Transformer model, despite its strength, exhibits larger deviations under rapid changes, reflecting the challenges deep learning models face in capturing complex temporal patterns on their own.

In conclusion, the proposed hybrid model’s ability to closely follow actual wind speed trends, even during rapid changes, makes it a reliable tool for wind speed forecasting. The visual comparison of predicted versus actual values across different models provides a clear assessment of their performance, demonstrating the hybrid model’s superior accuracy and robustness.

### Discussion

The results highlight that the proposed hybrid model surpasses traditional and deep learning-based methods in wind speed forecasting, offering a robust framework through the integration of WT for feature extraction, a Transformer-based encoder-decoder for capturing long-term dependencies, and XGBoost for ensemble learning. The incorporation of CBO for hyperparameter optimization further enhances the model’s efficiency and convergence speed. A key strength of the model lies in its ability to capture both short-term fluctuations and long-term trends in wind speed data, making it highly applicable for renewable energy optimization, aviation safety, and meteorological predictions. By combining the strengths of Transformer and XGBoost, the ensemble strategy improves generalization and robustness, while the use of CBO with Adam significantly boosts computational efficiency, enhancing its practicality for real-time applications. However, despite its high accuracy, the model’s computational complexity may pose challenges in scenarios with strict latency requirements, suggesting a need for future exploration of lightweight architectures or model compression techniques. Additionally, extending the model to include additional meteorological features, such as temperature, humidity, and pressure, could further refine forecasting accuracy.

While the proposed CBOTran-XGBoost model demonstrates strong forecasting accuracy, it has some limitations. The Transformer component introduces a relatively large parameter count, leading to higher memory usage and longer training times. Additionally, model performance is sensitive to hyperparameter settings, requiring optimization via CBO, which adds complexity.

To address these issues, we propose several mitigation strategies for future work:

- Apply post-training quantization and pruning to reduce model size and speed up inference.
- Explore lightweight Transformer variants (e.g., TinyTransformer, DistilTransformer) to improve efficiency.
- Investigate AutoML or NAS-based tuning to minimize manual intervention.

These enhancements aim to support real-time deployment in edge environments and improve the model’s scalability and practicality.

## Conclusion and future work

The proposed hybrid model, which integrates WT decomposition, a Transformer-based encoder-decoder model, and an ensemble strategy combining Transformer outputs with XGBoost, demonstrates superior performance in wind speed forecasting for the tested Egyptian dataset. The model achieves the lowest MAE, RMSE, and MAPE, along with the highest R² Score, outperforming all baseline and state-of-the-art models. The integration of CBO for hyper parameter optimization further enhances the model’s efficiency and convergence speed, making it a practical solution for real-world applications. Future work will focus on improving computational efficiency and extending the model to incorporate additional meteorological features for even greater forecasting accuracy. A limitation of this study is that the model was evaluated using data from a single location. While the results are promising, they may not fully reflect the variability present in other geographic regions. Future work will include testing the model on multi-site datasets to better assess its generalizability and robustness across different environmental conditions.

## Electronic supplementary material

Below is the link to the electronic supplementary material.

Supplementary Material 1

## Figures and Tables

**Algorithm 1:**
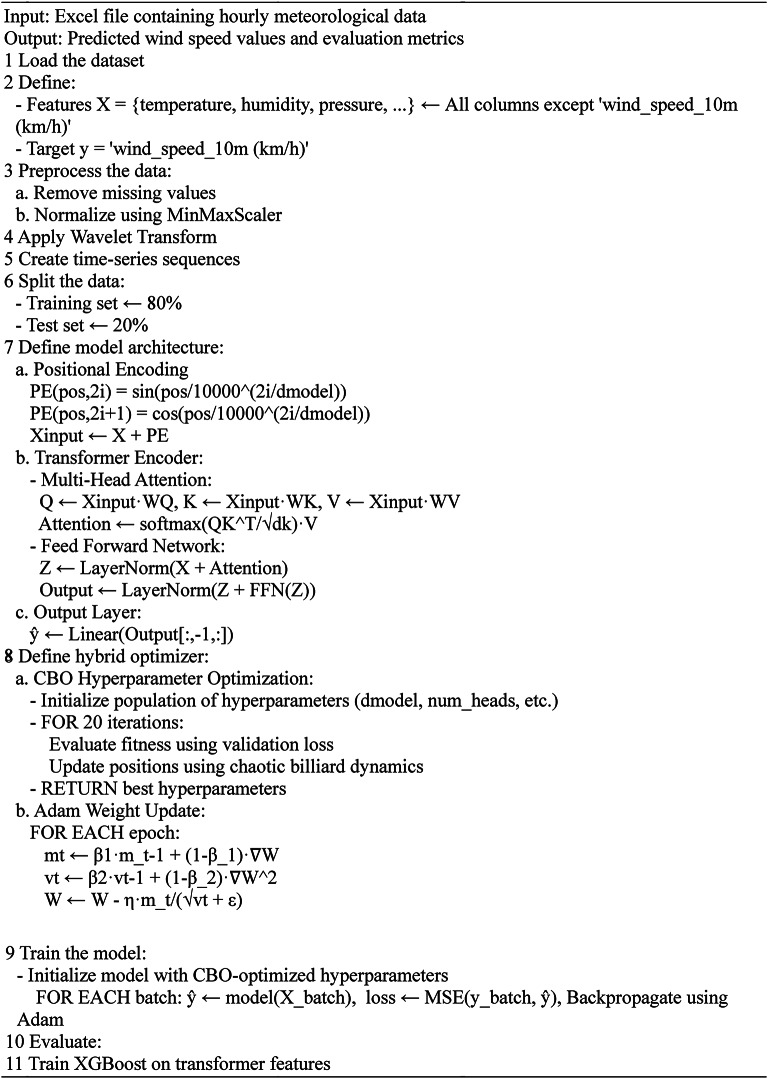
CBOTran-XGBoost hybrid model for wind speed srediction

## Data Availability

The data that support the findings of this study are available from the Open-Meteo historical weather API. The dataset includes hourly weather data for five cities in Egypt from 2020 to 2024. This dataset can be accessed through the Open-Meteo website at https://open-meteo.com. For specific queries or requests related to the data, please contact [Open-Meteo Support/Contact Information if available].Corresponding authors email: reham.elenani@eng.suez.edu.eg.

## List of symbols

WT: Wavelet transform
EMD: Empirical mode decomposition
SSA: Singular spectrum analysis
CEEMDAN: CEEMD with adaptive noise
ML: Machine learning
LSTM: Long short-term memory
GRU: Gated recurrent unit
NARX: Nonlinear autoregressive exogenous
GBRT: Gradient boosted regression trees
FFN: Feed-forward network
CBO: Chaotic billiards optimizer
CSO: Chicken swarm optimization
MOO: Multi-objective optimization
MSE: Mean squared error
MAPE: Mean absolute percentage error
EVS: Explained variance score
DWT: Discrete wavelet transform
VMD: Variational mode
EWT: Empirical wavelet transform
STC: Spatio-temporal correlation
DL: Deep learning
RNN: Recurrent neural network
ARIMA: Auto-regressive integrated moving average
MHA: Multi-head attention
XGBoost: Extreme gradient boosting
BSA: Backtracking search algorithm
GA: Genetic algorithm
MAE: Mean absolute error
RMSE: Root mean squared error
R^2^: Coefficient of determination
PE: Positional encoding
W_t_: Wind speed at time t
H_t_: Humidity at time t
Dj(t): Detail coefficients
Xt: Input feature vector
Q, K, V: Query, Key, Value matrices
d_FFN_: FFN layer size
PE: Positional encoding
X_input_: Transformer input
m_t_, v_t_: Adam moment estimates
ε: Stability constant
n: Total data points
Var(y): Variance of data
B_{n,m}_: Position of ball n,m
Ω: Cue movement coeff.
Iter: Current iteration
T_t_: Temperature at time t
P_t_: Pressure at time t
A_j_(t): Approximation coefficients
Y_t_: Target (wind speed)
d: Feature dimension
h: Number of attention heads
X': Normalized input
ŷt: Predicted wind speed
η: Learning rate
θ: Model parameters
$\bar{y}~~$: Mean actual value
PR: Accuracy rate (CBO)
PK: Pocket position (BOA)
a: Acceleration (BOA)
iter_max_: SMax iteration

## References

[1] Wang, J., Wu, C. & Niu, T. A novel system for wind speed forecasting based on multi-objective optimization and echo state network. *Sustainability* **11**, 526. 10.3390/su11020526 (2019).

[2] Chen, Y. et al. A novel combined model based on echo state network for multi-step ahead wind speed forecasting: A case study of NREL. *Energy. Conv. Manag.* **179**, 13–29. 10.1016/j.enconman.2018.10.068 (2019).

[3] Li, D., Jiang, F., Chen, M. & Qian, T. Multi-step-ahead wind speed forecasting based on a hybrid decomposition method and Temporal convolutional networks. *Energy* **238**, 121981. 10.1016/j.energy.2021.121981 (2022).

[4] Peng, T., Zhang, C., Zhou, J. & Nazir, M. S. Negative correlation learning-based RELM ensemble model integrated with OVMD for multi-step ahead wind speed forecasting. *Renew. Energy*. **156**, 804–819. 10.1016/j.renene.2020.03.168 (2020).

[5] Liu, H. & Chen, C. Multi-objective data-ensemble wind speed forecasting model with stacked sparse autoencoder and adaptive decomposition-based error correction. *Appl. Energy*. **254**, 113686. 10.1016/j.apenergy.2019.113686 (2019).

[6] Niu, X. & Wang, J. A combined model based on data preprocessing strategy and multi-objective optimization algorithm for short-term wind speed forecasting. *Appl. Energy*. **241**, 519–539. 10.1016/j.apenergy.2019.03.097 (2019).

[7] Liu, X., Lin, Z. & Feng, Z. Short-term offshore wind speed forecast by seasonal ARIMA - A comparison against GRU and LSTM. *Energy* **227**, 120492. 10.1016/j.energy.2021.120492 (2021).

[8] Pliego Marugán, A., García Márquez, F. P., Pinar Perez, J. M. & Ruiz-Hernández, D. A survey of artificial neural network in wind energy systems. *Appl. Energy*. **228**, 1822–1836. 10.1016/j.apenergy.2018.07.084 (2018).

[9] Liu, H., Li, Y., Duan, Z. & Chen, C. A review on multi-objective optimization framework in wind energy forecasting techniques and applications. *Energy. Conv. Manag.* **224**, 113324. 10.1016/j.enconman.2020.113324 (2020).

[10] Jiang, Z., Che, J. & Wang, L. Ultra-short-term wind speed forecasting based on EMD-VAR model and Spatial correlation. *Energy. Conv. Manag.* **250**, 114919. 10.1016/j.enconman.2021.114919 (2021).

[11] Fu, W. et al. Multi-step ahead short-term wind speed forecasting approach coupling variational mode decomposition, improved beetle antennae search algorithm-based synchronous optimization and Volterra series model. *Renew. Energy*. **179**, 1122–1139. 10.1016/j.renene.2021.07.119 (2021).

[12] Qu, Z., Mao, W., Zhang, K., Zhang, W. & Li, Z. Multi-step wind speed forecasting based on a hybrid decomposition technique and an improved back-propagation neural network. *Renew. Energy*. **133**, 919–929. 10.1016/j.renene.2018.10.043 (2019). 10.1007/s11356-022-19388-4 35220530

[13] Xiang, L., Li, J., Hu, A. & Zhang, Y. Deterministic and probabilistic multi-step forecasting for short-term wind speed based on secondary decomposition and a deep learning method. *Energy. Conv. Manag.* **220**, 113098. 10.1016/j.enconman.2020.113098 (2020).

[14] Zhang, C., Zhou, J., Li, C., Fu, W. & Peng, T. A compound structure of ELM based on feature selection and parameter optimization using hybrid Backtracking search algorithm for wind speed forecasting. *Energy. Conv. Manag.* **143**, 360–376. 10.1016/j.enconman.2017.04.007 (2017).

[15] Liu, H., Yang, R., Wang, T. & Zhang, L. A hybrid neural network model for short-term wind speed forecasting based on decomposition, multi-learner ensemble, and adaptive multiple error corrections. *Renew. Energy*. **165**, 573–594. 10.1016/j.renene.2020.11.002 (2021).

[16] Moreno, S. R., Mariani, V. C. & dos Santos Coelho, L. Hybrid multi-stage decomposition with parametric model applied to wind speed forecasting in Brazilian Northeast. *Renew. Energy*. **164**, 1508–1526. 10.1016/j.renene.2020.10.126 (2021).

[17] Karasu, S., Altan, A., Saraç, Z. & Hacıoğlu, R. Estimation of fast varied wind speed based on NARX neural network by using curve fitting. *IJEAT* **4** (3), 137–146 (2017).

[18] Chen, Y., Zhang, S., Zhang, W., Peng, J. & Cai, Y. Multifactor spatio-temporal correlation model based on a combination of convolutional neural network and long short-term memory neural network for wind speed forecasting. *Energy. Conv. Manag.* **185**, 783–799. 10.1016/j.enconman.2019.02.018 (2019).

[19] Memarzadeh, G. & Keynia, F. A new short-term wind speed forecasting method based on fine-tuned LSTM neural network and optimal input sets. *Energy. Conv. Manag.* **213**, 112824. 10.1016/j.enconman.2020.112824 (2020).

[20] Ehsan, M. A. et al. Wind speed prediction and visualization using long short-term memory networks (LSTM). In 2020 10th International Conference on Information Science and Technology (ICIST) (pp. 1–6). IEEE. (2020). 10.1109/ICIST48822.2020.9230953

[21] Gan, Q., Gong, L., Hu, D., Jiang, Y. & Ding, X. A hybrid missing data imputation method for batch process monitoring dataset. *Sens. (Basel)*. **23** (21), 8678. 10.3390/s23218678 (2023). 10.3390/s23218678 PMC10650138 37960379

[22] Rhif, M., Ben Abbes, A., Farah, I. R., Martínez, B. & Sang, Y. Wavelet transform application for/in non-stationary time-series analysis: A review. *Appl. Sci.* **9** (7), 1345. 10.3390/app9071345 (2019).

[23] Pospíchal, J., Kubovčík, M. & Dirgová Luptáková, I. Solar irradiance forecasting with transformer model. *Appl. Sci.* **12** (17), 8852. 10.3390/app12178852 (2022).

[24] Lim, B., Arık, S. Ö., Loeff, N. & Pfister, T. Temporal fusion Transformers for interpretable multi-horizon time series forecasting. *Int. J. Forecast.* **37** (4), 1748–1764 (2021).

[25] Brandon, W., Mishra, M., Nrusimha, A., Panda, R. & Ragan-Kelley, J. *Reducing Transformer key-value Cache Size with cross-layer Attention* (Advances in Neural Information Processing Systems (NeurIPS, 2024).

[26] Almaafi, A., Bajaba, S. & Alnori, F. Stock price prediction using ARIMA versus XGBoost models: the case of the largest telecommunication company in the middle East. *Int. J. Inf. Technol.* **15**, 1813–1818. 10.1007/s41870-023-01260-4 (2023).

[27] Reyad, M., Sarhan, A. M. & Arafa, M. A modified Adam algorithm for deep neural network optimization. *Neural Comput. Appl.* **35**, 14041–14056. 10.1007/s00521-023-08568-z (2023).

[28] Kaveh, A., Khanzadi, M. & Rastegar Moghaddam, M. Billiards-inspired optimization algorithm; a new meta-heuristic method. *Structures* **27**, 1722–1739. 10.1016/j.istruc.2020.07.058 (2020).

[29] Landry, J-F. & Dussault, J-P. AI optimization of a billiard player. *J. Intell. Robot Syst.* **50**, 399–417. 10.1007/s10846-007-9172-7 (2007).

[30] Ma, Z. Chaotic populations in genetic algorithms. *Appl. Soft Comput. J.* **12** (8), 2409–2424. 10.1016/j.asoc.2012.03.001 (2012).

[31] Dos Coelho, L. S. & Alotto, P. Multiobjective electromagnetic optimization based on a nondominated sorting genetic approach with a chaotic crossover operator. *IEEE Trans. Magn.* **44** (6), 1078–1081. 10.1109/TMAG.2007.916027 (2008).

[32] Plevris, V., Solorzano, G., Bakas, N. P. & Ben Seghier, M. E. A. Investigation of performance metrics in regression analysis and machine learning-based prediction models. *J. Appl. Sci. Eng.* **25** (3), 631–646 (2022).

[33] Gad, A. G. Particle swarm optimization algorithm and its applications: A systematic review. *Arch. Computat Methods Eng.* **29**, 2531–2561. 10.1007/s11831-021-09694-4 (2022).

[34] Anand, M. M. S., Aravindan, S. & Rao, P. V. Genetic algorithm and its applications to mechanical engineering: A review. *Procedia Comput. Sci.* **57**, 765–774. 10.1016/j.procs.2015.07.485 (2015).

[35] Li, X., Li, K., Shen, S. & Tian, Y. Exploring time series models for wind speed forecasting: A comparative analysis. *Energies* **16** (23), 7785. 10.3390/en16237785 (2023).

[36] Fan, Y. & Lei, W. Wind speed prediction based on gradient boosting decision tree. In* Proceedings of the 2022 International Conference on Big Data, Information and Computer Network (BDICN), Sanya*, China, 93–97.(2022).10.1109/BDICN55575.2022.00025

[37] Elnaghi, B. E., Ismaiel, A. M. & El Abdel-Kader, S. Validation of energy Valley optimization for adaptive fuzzy logic controller of DFIG-based wind turbines. *Sci. Rep.* **15**, 711. 10.1038/s41598-024-82382-y (2025). 39753637 10.1038/s41598-024-82382-y PMC11699151

[38] Elnaghi, B. E., Abelwhab, M. N., Ismaiel, A. M. & Mohammed, R. H. Solar hydrogen variable speed control of induction motor based on chaotic billiards optimization technique. *Energies* **16** (3), 1110. 10.3390/en16031110 (2023).

